# Exploring the potential of ultrasound: Novel approaches to collagen extraction, modifications, and polypeptide preparation^☆^

**DOI:** 10.1016/j.ultsonch.2025.107504

**Published:** 2025-08-11

**Authors:** Yongjun Zhao, Mingming Zhong, Shuixiu Pang, Ping Liu, Yufan Sun, Qiufang Liang, Kai Yu, Junxia Wang, Abdur Rehman, Arif Rashid, Haile Ma, Xiaofeng Ren

**Affiliations:** a School of Food and Biological Engineering, Jiangsu University, 301 Xuefu Road, Zhenjiang, Jiangsu 212013, China; b Institute of Food Physical Processing, Jiangsu University, 301 Xuefu Road, Zhenjiang, Jiangsu 212013, China; c Zhongke Zhigu International Pharmaceutical Biotechnology (Guangdong) Co., Ltd, Guikeng Village, Chuangxing Avenue, Gaoxin District, Qingyuan, Guangdong 511538, China; d School of Food Science and Technology, Jiangsu Agri-animal Husbandry Vocational College, No 8 East Phoenix Road, Taizhou, Jiangsu 225300, China

**Keywords:** Collagen, Ultrasound-assisted processing, Extraction, Modification, Techno-functional properties, Biological activities

## Abstract

Collagen, a protein of considerable abundance in animal tissues, is increasing recognized in food industry for its potent nutritional properties and health benefits. Nevertheless, the suboptimal extraction rate and inefficiencies associated with conventional collagen processing posed significant barriers to its widespread application. Recent advances have spurred interest in the utilization of the ultrasound (US) technology as means to improve collagen extraction processes. The ultrasound can be presented as a highly effective method for auxiliary collagen extraction, characterized by substantial yield, enhanced extraction efficiency, and the preservation of the functional activity. Ultrasound treatment can facilitate improvement in both solubility and molecular structure of collagen, thereby promoting its functional properties. This review aims to elucidate ultrasound-assisted methods aimed at optimizing collagen extraction efficiency, while also exploring the intricate relationship between ultrasound processing parameters and collagen yield. Furthermore, it examines the correlation between collagen structural attributes and its functional efficacy. Moreover, the review delineates the advantages of the ultrasound-assisted methods in the extraction of collagen peptides and addresses the current challenges along with the perspective trends in ultrasonic applications. Notably, ultrasonic cavitation partially unfolds collagen, enhancing its efficacy when synergistically applied with other extraction techniques. Therefore, the advancement of ultrasonic equipment and the optimization of processing conditions are crucial for the innovation of collagen-based functional foods.

## Graphical abstract

**Figure:**
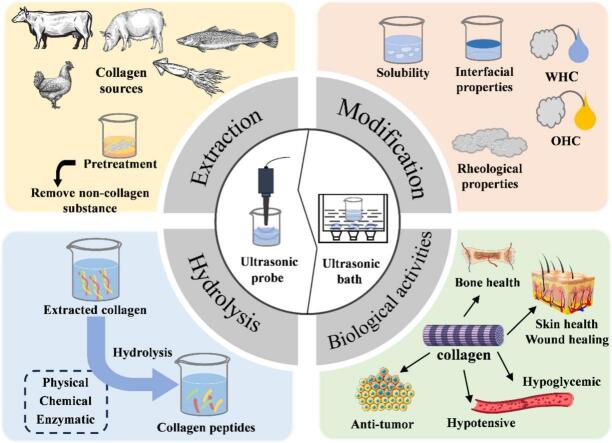

## 1 Introduction

Collagen is a fundamental and ubiquitous component of the extracellular matrix, constituting over 30 % of the total protein content in nearly all animal species [1]. It is predominantly found in connective tissues, with significant concentrations in ligaments, tendons, and skin [2]. The biological functions of collagen extend beyond mere structural support; it plays a critical role in the functionality of nearly all systems and organs within the human body. Consequently, collagen is an essential protein that is intricately linked to overall health through its physical and chemical properties [3].

Traditionally, collagen has been extracted from cattle and pig products, and poultry products are also used as an alternative collagen source. However, due to the influence of animal diseases and prohibitions of some cultures and religions, its use has decreased [4]. In contrast, collagen derived from aquatic organisms is gaining popularity, especially for marine collagen [5]. Despite the abundance of animal sources, the complexity and instability of its raw materials limit its application. Enhancing collagen’s functional properties through extraction and modification boosts its commercial value and improves its use as a high-value ingredient [6]. Moreover, the peptides produced by collagen have higher bioavailability and bioactivity and a wider range of applications [7].

In comparison to traditional chemical and enzymatic processing methods, physical processing tactics have gained popularity in the food industry owing to their cost-effectiveness, the absence of synthetic additives, and their environmentally friendly nature [8,9]. In recent years, researchers have explored innovative physical methods, including ultrasound, microwave treatment, high-voltage techniques, and pulsed electric fields, to evaluate their effects on the physicochemical and functional properties of proteins [10]. In these methods, ultrasound has gained particular research interest for extracting bioactive compounds, primarily attributed to its cavitation effects. Ultrasound changes collagen properties by inducing mechanical, physical, and chemical/biochemical changes in collagen through cavitation, thereby reducing processing time and improving efficacy. At the same time, ultrasonic treatment can cause obvious physical changes, making the microstructure of collagen more loose, more porous, and more uniform [11]. These alterations in microstructure can effectively accelerate the hydrolysis process of collagen peptides and improve hydrolysis efficiency [12].

This review examines recent advancements in ultrasonic technology related to the extraction, structural modification, and hydrolysis of collagen, emphasizing their influence on its techno-functional properties. The aim is to educate readers about collagen and ultrasound technology, ultimately broadening the use of ultrasound in collagen processing and encouraging its wider adoption in the food industry.

## 2 The natural properties of collagen

### 2.1 Collagen

Collagen is a ubiquitous protein found in the bones and skin of all animal species, and it constitutes the most abundant structural protein across the animal kingdom [13]. Collagen, a structural protein, is known for its durability, flexibility, and stability. The formation of the basement membrane and extracellular matrix is contingent upon a complex architecture of fibril and microfiber structures. Present in connective tissues such as skin, tendons, cartilage, and bone, collagen plays a crucial role in maintaining tissue integrity. Furthermore, collagen fibrils significantly enhance the stability of various organs and tissues by providing exceptional tensile strength.

**Fig. 1:**
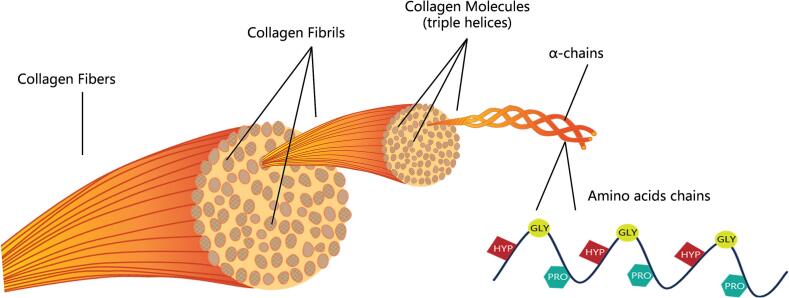
The structure of collagen.

The general structural feature of collagen is a right-handed triple helix structure formed by the supercoil of three parallel alpha chains, each of which adopts a left-handed conformation similar to polyproline II (PPII) **(**Fig. 1**)** [14]. In the 1950s, Ramachandran and Kartha used X-ray diffraction to propose the structure of collagen as a triple helix, which established the basis for a deeper understanding of collagen's functions and properties [15]. Researchers have identified 29 genetically distinct collagens to date. Each collagen shows significant variation in its amino acid sequence, structure, and performance, based on sequence homology and molecular structure [16]. Collagen, the most abundant and essential protein in the human body, is integral to the maintenance of health. Collagen-based materials have wide application prospects because of their availability and remarkable biocompatibility [17].

### 2.2 Collagen sources

Collagen sources are categorized into natural and synthetic groups. Natural collagen is sourced from animals, including mammals, fish, marine species, and birds [18]. Although natural collagen is widely used in market products, synthetic collagen is also produced [19]. Technological advancements enable collagen production through recombinant systems, involving bacteria, yeast, insects, plants, mammalian cells, and artificial fibrils [20,21]. Collagen in mammals such as bovine and porcine and collagen in marine fish are the most popular sources of animal collagen. Table 1 summarizes the properties of collagen from different sources. This study focuses on bovine, porcine, marine, and poultry collagen.

**Table 1:** The sources and characteristics of collagen.

Sources	Part	Structure and properties	Biological compatibility	Disadvantages	Ref.
Bovine	Skin, bone	• Type I and III collagen • High thermal stability • High mechanical strength	• Minimal immunogenicity • Significant biocompatibility • Rapid biodegradability	• The spread of various diseases such as FMD, TSE and BSE	[22,23,24]
Porcine	Skin, bone	• Types I and III collagen • Excellent thermal stability • High mechanical strength • Low molecular weight	• Allergies impact 2–4 % of treated individuals • Biocompatible, no foreign body reaction	• Risk of disease transmission (swine fever) • Religious restriction	[[13], [25], [26], [27]]
Aquatic	Bones, scales, skin and, skull	• Types I, II and IV collagen • Low water solubility, melting point, and viscosity	• Low inflammatory response • Less immunogenic action • No risk of spreading disease	• Harvest seasonal and regional effects • Complicated process • Time-consuming • Low yield	[28,[29], [30], [31]]
Poultry	Skin, bone	• Types I, II and III collagen • Better structural stability	• Low risk of animal disease transmission	• Religious restriction	[[25], [32], [33]]

#### 2.2.1 Mammal

The most abundant sources of commercial and high-cost collagen are pig skin, pork, bovine hide, cattle bones and other mammalian sources [2]. Bovine bones and skin, including those of cows, oxen, buffaloes, and cattle, are the primary sources of collagen [22]. Despite various tissue sources, collagen type III, solely used for research, is obtained from bovine skin. Unfortunately, bovine collagen lacks essential amino acids, rendering it an incomplete protein source [34]. The spread of dangerous diseases like mad cow disease, foot and mouth disease (FMD), bovine spongiform encephalopathy (BSE), and transmissible spongiform encephalopathies (TSE) has led researchers to look for safer collagen sources. Porcine collagen shares many properties with bovine collagen, including mechanical strength, thermal stability, and swelling ratio [35]. Porcine collagen is a highly cost-effective collagen source [36]. However, zoonosis, like bovine collagen, poses contamination risks, and religious restrictions prohibit the use of pigs [23]. Bovine, pig skin and bones are classified as traditional sources of type I collagen [37]. Comparative analysis reveals 85 %–92 % sequence similarity in their α-chain amino acid compositions, particularly in type I collagen isoforms. Crucially, both collagens maintain identical core triple-helical conformation stabilized by characteristic Gly-X-Y repeat sequences within their structural domains. As members of the same fibrillar collagen classification, these xenogeneic sources demonstrate equivalent hierarchical organization from molecular to supramolecular assemblies [38].

#### 2.2.2 Aquatic

Collagen derived from aquatic organisms is a kind of protein with wide bioactivity and application potential. It is mainly derived from marine life [39]. Marine collagen sources are categorized into those from vertebrates and invertebrates [28]. The Food and Drug Administration (FDA) has deemed marine collagen as generally recognized as safe (GRAS) and free from disease transmission risks [40]. Collagen type II is primarily derived from fish cartilage, comprising 90–95 % of the cartilage total protein content [41]. Collagen type V is present in marine invertebrates like mollusks and crustaceans. Mammalian collagen is restricted by diseases such as BSE and religious constraints. Consequently, aquatic collagen has gained attention as an ideal resource for product development [42].

#### 2.2.3 Poultry

Another source of collagen is found in the joints and skin of poultry byproducts, including the bones, feet, and skin of chickens and ducks [43]. Chicken neck is the source of collagen types I, II, III, and V, with type I being the most common. The sternal cartilage of chicken embryos contains type IX, whereas the epidermis has types I and III, and the muscle tissues include type IV [[32], [44], [45], [46]]. In terms of structure, biology, and socioeconomics, chicken collagen performs better than collagen from other sources. These benefits increase its appeal by allowing it to be used in a variety of sectors [47]. Moreover, poultry collagen is more widely accepted by consumers with diverse beliefs compared to bovine and porcine collagen.

#### 2.2.4 Recombinant collagen

Traditionally, collagen has been extracted from bovines, porcine, aquatic or poultry, but these processes are time-consuming and have different product quality due to differences in individual animals and the presence of animal viruses [48]. Recombinant collagen is a protein obtained by using human collagen cDNA fragments as the backbone gene, cloning the gene to the selected expression vector and converting it into an expression cell, and finally achieved by purification technology [49]. Recombinant collagen production predominantly utilizes four expression systems: microbial expression systems including prokaryotic (*E. coli*) and eukaryotic (yeast), plant expression systems, animal expression systems and insect baculovirus expression systems [50]. Table 2 summarizes the advantages and disadvantages of producing recombinant collagen in different expression systems.

**Table 2:** Advantages and disadvantages of recombinant collagen production in different expression systems [51].

Expression system	Advantages	Disadvantages
Prokaryotic (E. coli)	Simple genetic manipulation; low fermentation cost; short production cycle; high efficiency	Low necessary modification; endotoxin; low protein activity; lack of hydroxylation
Eukaryotic (yeast)	Higher safety; low fermentation cost; high yield	Difficult to produce heterologous collagen
Plants	Can be scaled; short production cycle; low cost; high safety	Long growth cycle; low yield; pollution of the environment
Animals	Stable expression and stable yield	Long expression cycle; high cost; easily contaminated by bacteria; low expression level
Insect baculovirus expression system	Low background interference; correct protein folding; high protein activity	High cost; long growth cycle; low expression level

## 3 Collagen extraction process

The efficiency and effectiveness of the collagen extraction process are critical, as they directly influence the properties of the extracted collagen. This process typically varies depending on the raw material used. The goal is to eliminate all non-collagen substances, restore collagen as the end product, and improve both the quantity and quality of collagen extraction [52]. Collagen extraction usually involves two basic stages: pretreatment and extraction. The main goal of pretreatment phase is to eliminate non-collagen proteins and pigments and improve the effectiveness of the extraction process. Throughout the collagen extraction process, it is essential to maintain the integrity of its triple helix structure to the greatest extent possible. Various extraction methods classify collagens into acid-soluble (ASC), salt-soluble (SSC), pepsin-soluble (PSC), and ultrasound-assisted collagen (UAC). The yield and physiochemical properties of extracted collagens differ based on the extraction method. A summary of different collagen extraction methods and their respective yields is presented in Table 3**.**

**Table 3:** The extraction technologies of collagen and their yield.

Sources	Part	Extraction methods	Yield	Ref.
Buffalo	Skin	ASC	Acid solubilized collagen was extracted from buffalo skin, yielding 1.8 % (based on wet skin weight)	[24]
Bovine	Bone	ASC, PSC, and Enzyme soluble collagen (ESC)	The yield of PSC (10.59 %) and ESC (12.18 %) was significantly increased compared with that of ASC (6.13 %)	[53]
Bovine	Skin	ASC and Acid‑enzyme soluble collagen (AESC)	The highest dry collagen content was from cow hides (75.13 %), followed bull hides at 74.45 %	[54]
Sharpnose stingray (*Dasyatis zugei*)	Skin	ASC and PSC	The yield of extracted PSC (34.84 ± 1.26 %) was higher than the extracted ASC (20.48 ± 4.41 %)	[55]
Golden carp (*Probarbus Jullieni)*	Skin	ASC, PSC, and Ultrasound-assisted extraction (UAE)	The ASC and PSC with ultrasound assisted had the yields of 81.53 and 94.88 %, with typical method showed the yields of 51.90 and 79.27 %	[56]
Tuna	Tendon	ASC, PSC, Vinegar-soluble collagen, and UAE	The ultrasonic assisted pepsin extraction method showed the highest yield of collagen (14.94 %)	[57]
Mackerel (*Scomber japonicus*)	Bone and skin	PSC	The PSC yields from mackerel bone and skin were 1.75 ± 0.07 and 8.10 ± 0.12 %	[58]
Bigeye tuna (*Thunnus obesus*)	Skin, scale, and bone	ASC and PSC	The yield of ASC and PSC in skin were 13.5 % and 16.7 %. The yields of PSC in scale and bone were 4.6 % and 2.6	[31]
Small-Spotted Catshark (*S. canicula*)	Skin	ASC	Under the optimal extraction conditions, the ASC yield was 61.24 %	[59]
Broiler chicken	Trachea	ASC, PSC, and UAE	After ultrasonic treatment, the collagen yield was increased to 1.58 % and 6.28 %, respectively. of 0.65 % and 3.10 %	[32]
Spent-hens	Bone	ASC, Alkali-soluble collagen (ALSC), and PSC	The extraction method had a significant effect on the yield of collagen, with the highest yield of PSC and the lowest of ALSC	[60]
Chicken	Sternal cartilage	PSC and UAE	The yield of ultrasound-treated pepsin soluble collagen-II significantly increased with longer treatment times, from 1.730 g to 3.367 g	[46]
Atlantic cod	Skin	DES; Mixing urea and propionic acid in a 1:2 ratio	Collagen yield of 2.2 ± 0.3 %	[61]
Chicken	Feet	Enzyme treatment (papain)	The highest yield was 32.16 % (w/w)	[62]
Salt brine Atlantic cod	Skin	SFE; Using water acidified with CO2; Temperature: 37 ◦C, Pressure: 50 bars	Extraction yield of 13.8 %	[63]

### 3.1 Pretreatment

Choosing the right pretreatment is crucial for removing impurities and improving collagen quality. Food sources contain non-collagenous proteins, lipids, pigments, and minerals alongside collagen, necessitating pretreatments to eliminate these compounds [64]. In the pre-treatment stage, the raw material is first classified, which facilitates subsequent cleaning, reduction of size, and removal of contaminants [65]. Reducing the size of the sample is necessary to facilitate subsequent pre-treatment steps, which aim to remove excess collagen, pigment, or fat. A typical method is alkaline pretreatment with sodium hydroxide (NaOH). In addition, demineralization with inorganic compounds like EDTA (ethylenediaminetetraacetic acid) is typically performed to enhance collagen extraction from bone, cartilage, and scales. Employ sodium chloride, sodium hydrochloride, and n-butanol to eliminate collagen dyes and fats [66]. Animal connective tissue contains cross-linked collagen, which undergoes pretreatment with diluted acids or bases before extraction [67]. In summary, acid pretreatment serves to disrupt non-covalent bonds within the collagen structure. Conversely, alkaline pretreatment using sodium hydroxide (NaOH) enhances swelling and facilitates collagen extraction by improving the transfer of the tissue matrix [68].

### 3.2 Traditional extraction method

Collagen is commonly extracted by disrupting the helix structure through four primary methods: acid extraction, alkali extraction, salt extraction, and enzyme extraction [67]. The fundamental principle of collagen extraction is based on the use of ions within various media solutions to penetrate the molecular structure of collagen. This interaction generates an osmotic pressure differential between the interior and exterior of the collagen molecules. This process leads to further swelling or dissolution. Subsequently, a salt solution, such as sodium chloride or ammonium sulfate, is employed for salting out, followed by precipitation and dialysis purification, ultimately achieving the goal of separation [69,70].

#### 3.2.1 Acid extraction

Collagen proteins exhibit fibrous characteristics and demonstrate reduced solubility in water compared to acidic environments. Consequently, the extraction of collagen can be improved through the use of an acidic solution, which disrupts the bonds between collagen molecules. Acid-soluble extraction typically employs organic acids such as acetic, citric, and lactic, as well as inorganic acids including hydrochloric and nitric. In this extraction method, organic acids are preferred due to their effectiveness in dissolving non-crosslinked collagens and disrupting interstrand cross-links [71]. Collagen extracted and prepared under organic acidic conditions is referred to as acid-soluble collagen (ACS). The inability of collagen from diverse sources to fully dissolve in acidic media results in a significantly low yield [60].

#### 3.2.2 Alkali extraction

Alkali extraction refers to the use of specific concentrations of alkali under specific external conditions to extract proteins [72]. The commonly used treatment agents in alkali treatment are sodium hydroxide and sodium carbonate, among which the extraction effect is better with sodium hydroxide, and the extraction speed is also faster [73]. However, alkali extraction is easy to denature collagen, resulting in the destruction of the secondary structure, and there is a risk of racemization, so it is generally not used [74].

#### 3.2.3 Salt extraction

Collagen exhibits solubility in saline solutions, thereby necessitating the use of such solutions during the extraction process. Typical neutral saline solutions include sodium chloride, citrates, and phosphates [65]. Collagen dissolved in salt water is referred to as salt-soluble collagen (SSC). It has been reported that 0.45 M NaCl at a 1:100 (w/v) ratio, stirred continuously for 24 h, can extract salt soluble collagen from Amur sturgeon (*Acipenser schrencki*) cartilage [75]. However, due to the comparatively lower solubility of collagen in saline solutions relative to other proteins, the utilization of this method for collagen extraction has encountered certain constraints.

#### 3.2.4 Enzymatic extraction

The method uses pepsin, trypsin, papain, and other collagenases in combination with specific conditions and pH to extract collagen at maximum yield. Collagens digested by pepsin are called pepsin-soluble collagen (PSC). In actual experimental operations, most researchers use the method of combining enzymes and organic acids to extract collagen, such as pepsin and acetic acid. For instance, collagen was extracted from mackerel bone and skin utilizing the pepsin enzyme, employing 0.57 M acetic acid with 0.1 % (w/v) pepsin over a duration of 72 h at a 1:10 (w/v) ratio. The mixture was then centrifuged and subsequently re-extracted using the identical procedure [58]. The triple helix structure of collagen obtained by enzyme extraction can remain intact, which is an effective extraction method. However, the enzymatic breakdown of collagen is influenced by a multitude of factors. Specifically, it is essential to sustain optimal temperature and pH values consistently during the entire process.

### 3.3 New extraction method

Although enzymatic and acid extraction techniques are frequently employed, soluble collagen chains can be severely broken down by additional environmental conditions, including higher temperatures and longer processing times. In order to increase collagen synthesis through the use of economical and less hazardous extraction methods, several researchers are working on the development of novel and ecologically friendly extraction techniques [76]. Currently, researchers are employing an increasing number of novel extraction techniques for protein extraction [77]. Ultrasonic-assisted extraction, a novel technology, offers several advantages over traditional techniques, such as ease of use, safety, environmental friendliness, quick processing time, and economic feasibility [78]. Compared to acid and gastric protein extraction, ultrasonic-assisted extraction improves collagen extraction efficiency (yield), especially in a short amount of time. The role and influence of ultrasonic technology in collagen extraction are described below.

#### 3.3.1 Ultrasound-assisted collagen

Ultrasound, characterized by high-frequency waves ranging from 20 to 1000 kHz, exceeds the threshold of human hearing, which is approximately 16 kHz [79]. Low-frequency (20–100 kHz), high-intensity ultrasound (10–1000 W/cm^2^) is commonly utilized for diffusion, extraction, and modification within the food industries [80]. It is employed as a tool for non-invasive and non-destructive analysis of food products during processing and storage. In recent years, ultrasound has demonstrated efficacy in extracting bioactive ingredients, improving extraction rates while maintaining extract quality with minimal or no impact [81,82]. Conventional techniques for collagen separation require significant time investment and result in increased residual tissue along with elevated levels of insoluble collagen content [83,56]. The ultrasound technique is employed for collagen extraction as an alternative to traditional methods, with the objective of minimizing processing time and improving yield [84].

Research suggests that ultrasound can be used directly as a pretreatment or extraction aid [85]. Ultrasound can induce physical changes in collagen fibrils, making their structure loose and porous, thus facilitating acid and enzyme treatment, which greatly helps to shorten the extraction time compared to traditional methods [28]. Compared with the traditional acid method, ultrasonic-assisted extraction of collagen can reduce the acetic acid concentration and increase the yield. Similarly, Zou et al. extracted acid-soluble collagen from soft-shelled turtles (*Pelodiscus sinensis*) calipash using ultrasound-assisted methods, showing a higher collagen yield (16.30 %) compared to other typical methods [86]. Ultrasound significantly impacts enzyme extraction of collagen by enhancing the dispersion of large enzyme aggregates and facilitating the opening of collagen fibrils, thereby promoting the transport of enzyme molecules to the surface of collagen fibrils [83]. Other studies suggest that ultrasound extraction can break hydrogen bonds between collagen chains, harming both the collagen proteins and the enzymes used to isolate them [87]. The ultrasound-assisted extraction (UAE) method demonstrated improved extractability of pepsin-soluble and acid-soluble collagens in a shorter time [88]. Although ultrasound assistance enhances collagen quality and quantity, maintaining proper parameters is essential. Under optimal ultrasonic conditions, collagen extraction time is reduced, and both quality and quantity are improved.

#### 3.3.2 Other extraction methods

Microwaves, a form of electromagnetic radiation used in microwave cooking, disrupt cell and tissue structure, facilitating extraction [89]. Microwaves penetrate deeply in tissue. Microwave assistance has been found to speedup acid and enzyme action compared to equivalent treatments without the microwaves [90]. Deep eutectic solvents (DES) are a type of ionic liquid formed by the combination of a hydrogen bond acceptor, such as a quaternary ammonium salt, and a hydrogen bond donor, such as a carboxylic acid or amine [91]. These solvents have been utilized in the extraction of a wide range of bioactive compounds from natural sources due to their low toxicity, biodegradability, and ability to solubilize a variety of compounds [92,93]. For example, collagen extraction from fish processing waste has been achieved using DES-based methods, highlighting the versatility of DES in collagen recovery from different waste streams [94]. Supercritical fluids are substances that are maintained at a temperature and pressure above their critical point, resulting in unique solvent properties. In the food industry, SFE has been applied in the extraction of essential oils, fats, and bioactive compounds from various natural sources [95,96]. The process of using carbon dioxide as a green solvent under supercritical conditions to successfully cover the skin and bones of striped catfish, collagen/gelatin, showed that the highest yields of 36.85 % and 8.10 % were obtained from the skin and bone, respectively [97].

## 4 Factors affecting the extraction effect of UAE

**Fig. 2:**
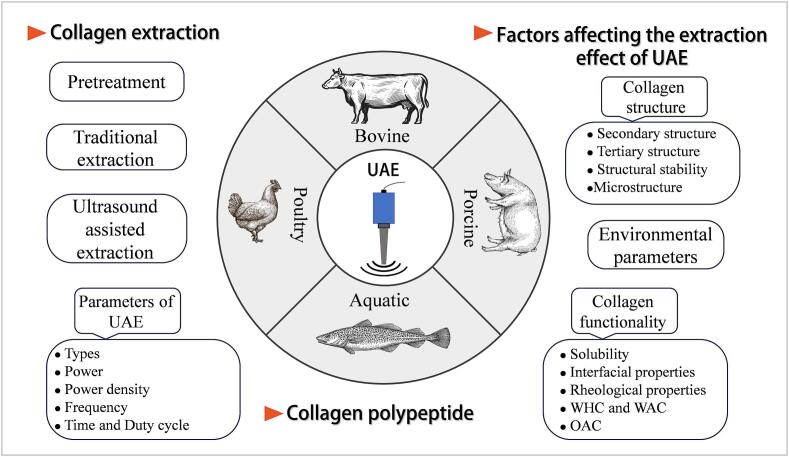
Diagram of the influence of ultrasonic-assisted technology on collagen.

Ultrasound-assisted extraction (UAE) of collagen is an effective extraction method that uses the cavitation effect of ultrasound to destroy tissue structure and release collagen. Several factors influence collagen extraction by ultrasound, including the source of collagen, the concentration of collagen in the solution, the parameters of ultrasound, and the treatment conditions [98,99]. Fig. 2 shows the impact of ultrasound-assisted technology on collagen.

### 4.1 Parameters of UAE

#### 4.1.1 Two classifications of UAE

Currently, there are primarily two kinds of ultrasonic equipment utilized in the UAE: bath-type ultrasonic and probe-type ultrasonic. Additionally, UAE equipment is categorized into direct and indirect extraction devices [79]. Probe-type ultrasonic equipment, a direct extraction method, is commonly used for processing small-volume samples. Ultrasonic baths, on the other hand, are indirect extraction tools for handling large quantities of samples. Under identical conditions, probe ultrasonics produce a more pronounced effect compared to ultrasonic baths [100]. Placement of the transducer plays a vital role in determining extraction yields. It is important for extraction efficiency, process intensification and energy losses. Transducers can be placed on either side of the extraction vessel, so ultrasonic waves will be transmitted through the outer wall of the extraction vessel. The major advantage of this arrangement is that transducers are not in direct contact with the sample, but significant losses of acoustic energy occur to vessel and surroundings. When transducers are in direct contact with the sample in the presence of a suitable solvent, extraction efficiency is enhanced, whilst minimizing acoustic energy losses. A probe-type system for UAE allows better extraction yields than ultrasonic bath system. With an ultrasonic probe, ultrasonic energy is applied directly to the sample and energy losses are minimal [101,102]. However, erosion of probes during extraction may leave residues in the extract. Additionally, chemical reactions between the probe material and the sample may cause corrosion, potentially damaging the probe over time [32]. Conversely, an ultrasonic bath has the advantage of preventing cross-contamination between consecutive experiments, unlike probe ultrasonics. Zou et al. used the probe-type ultrasonic equipment to extract acid-soluble collagen by UAE from soft-shelled turtle calipash. Results indicated a 16.3 % increase in collagen content with UAE compared to conventional extraction [103]. Ata et al. used an ultrasonic bath to extract pepsin-soluble collagen from lamb feet. The research determined that the optimal extraction condition for collagen by ultrasonic treatment was 25 °C/60 min [104]. Ata et al. applied ultrasonic baths (USBs) (frequency 35 kHz, power 140 W, time 45 min) and ultrasonic probes (USPs) (frequency 24 kHz, power 400 W, time 10 min) to extract collagen-I from lamb feet (LF) and found that compared to ultrasonic bath treatment and conventional extraction methods, USPs treatment significantly increased the collagen content of the extract (*p* < 0.05) [105]. Hence, generalizing that the probe-based systems are superior to ultrasonic baths may not be appropriate. However, in terms of process efficiency and energy losses, the ultrasonic probe-based system can be preferred for extraction purposes.

#### 4.1.2 Power of ultrasonic

The power of ultrasonic waves is directly related to their amplitude. Increased amplitude leads to greater power and stronger forces, improving extraction yield and efficiency [106]. Collagen yield from sea bass skin rose with increasing ultrasonic amplitude. Additionally, 20 % collagen can be extracted in 1.5 h at 80 % amplitude by an ultrasonic processor (VCX 750; Sonics & Materials,Newtown, CT), reducing extraction time by 16 times compared to non-ultrasonic methods [107]. Furthermore, the high-intensity or extended application of ultrasonic waves may lead to protein denaturation. For example, Petcharat and coworkers examined the effect of ultrasound on collagen extraction across various amplitudes (20–80 %), observing a rise in collagen yield (from 27.18 % to 57.35 %) from clown featherback (*Chitala ornata*) skin. The result suggested the co-extraction of other components from skin matrix since the high power of ultrasonication destructed skin matrix at the high degree. This caused the dilution effect and lowered hydroxyproline content of resulting collagen. While the molecular structure remained intact, reductions in hydroxyproline content and compromised collagen purity were noted [74]. Therefore, it is crucial to optimize power parameters in collagen extraction and select the appropriate power according to the characteristics of the sample.

#### 4.1.3 Power density of ultrasonic

Ultrasonic power density refers to the ultrasonic energy input per unit area, which is an important parameter in the process of ultrasonic extraction. Power density more accurately reflects ultrasonic intensity than ultrasonic power [108]. In general, the increase of ultrasonic intensity (UI) leads to a proportional rise in acoustic cavitational effects, thereby increasing the extraction rate of collagen [79]. However, a rise in UI leads to an increase in the number of bubbles. When it exceeds a certain limit, it will cause collisions and reduce the destructive effect of cavitation bubbles, reducing the cavitation effect. In addition, a large number of cavitation bubbles hinder energy transfer and reduce extraction efficiency [109,110]. For instance, Kitsanapong et al. studied the impact of ultrasound on collagen yield from broiler chicken trachea. UAE increased the yield of PSC; when power density was between 9.80 and 17.46 W∙cm^−2^, yield increased, but no further increase occurred from 17.46 to 27.56 W∙cm^−2^ [32].

#### 4.1.4 Frequency of ultrasonic

**Fig. 3:**
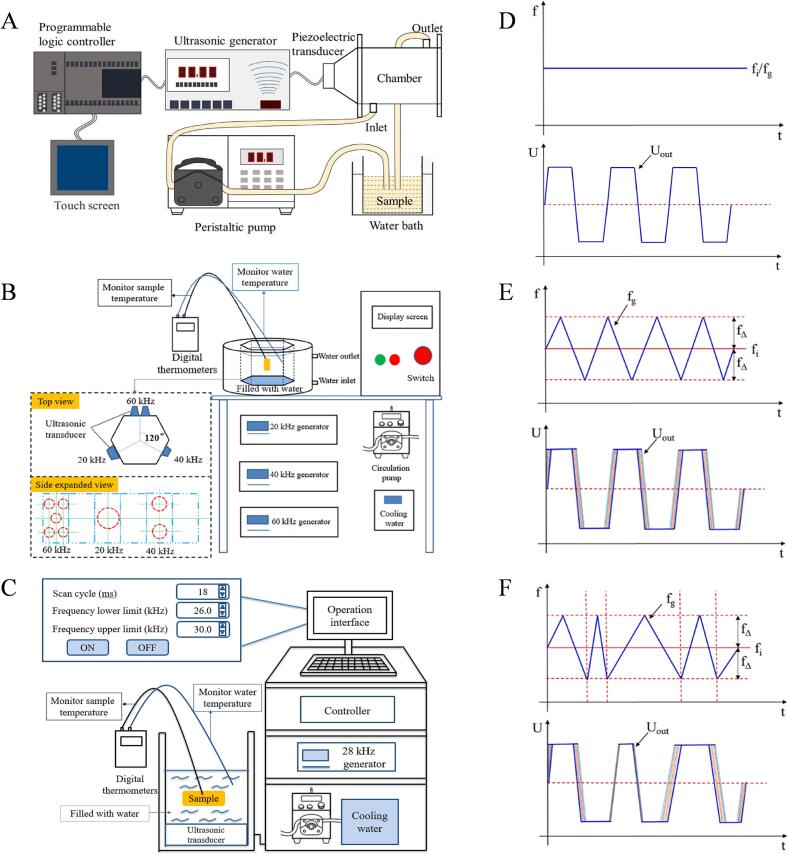
Schematic diagrams for single-frequency (A), multi-frequency (B), and sweep frequency (C) ultrasonic equipment. Frequency curves and output waveforms for single-frequency (D), multi-frequency (E), and random-sweeping frequency (F) modes.

Ultrasonic waves exceeding 20 kHz efficiently extract bioactive components from natural products [111]. Numerous investigations frequently utilize the most common frequencies, ranging from 20 kHz to 100 kHz, for protein extraction [112]. The mechanical stress on proteins is determined by the power and frequency of ultrasonic waves, which in turn affects the extraction rate and extent [113]. As an example, Akram and his group utilized ultrasound at a frequency of 20–25 kHz to extract collagen from chicken sternal cartilage [45]. The frequency range utilized in the UAE is contingent upon the various extraction materials employed. Numerous studies indicate that a low frequency range of 20–40 kHz is optimal for collagen extraction [79]. Researchers have been increasingly interested in the use of various ultrasonic devices in food preparation in recent years. Combinations of various ultrasonic frequencies have been shown to enhance effectiveness in numerous food processing applications [114]. Among them, dual-frequency or multi-frequency ultrasound can produce more complex and wider spectrum waveforms than single-frequency ultrasound, effectively preventing the formation of standing waves. Therefore, it shows a significantly greater comprehensive effect and leads to a stronger and more uniform protein extraction effect. Fig. 3 illustrates the ultrasonic frequency modes employed, which include single-frequency ultrasound (operating at one frequency), multi-frequency ultrasound (operating at multiple frequencies simultaneously), and the sweep mode of the same ultrasonic generator.

#### 4.1.5 Duty cycle of ultrasonic

The ultrasonic duty cycle is the ratio of working to stopping time within a specific period. It indirectly affects extraction results by influencing exposure time and temperature. At the same time, duty cycle also affects the wear of ultrasonic equipment, energy consumption, and extraction costs. For example, Shark et al. studied the impact of ultrasonic-assisted extraction on sharp-nosed stingray skin collagen. In order to avoid overheating during ultrasound treatment, ultrasound was used in pulse mode, operating for 5 s followed by 5 s of rest. However, few researchers have focused on the ultrasound duty cycle. The study found that the effect of a duty cycle on yield depends on treatment intensity and duration.

#### 4.1.6 Time of extraction

The effects of ultrasonic cavitation and mechanical oscillation over time contribute to the increase in collagen yield [71]. The time required for collagen extraction is affected by transfer rates. The diffusion process, evolving over time, plays a crucial role in governing the extraction. For instance, Pezeshk et al. found that ultrasound markedly enhances the collagen extraction rate over time when compared to controls. The highest collagen yield of 14.53 % was achieved after 25 min of ultrasound treatment [115]. In a similar manner, the collagen concentration of the PSC with the ultrasound-assisted process rose with the increase in time [104]. In another interesting investigation, Heidari and co-workers extracted collagen from yellowfin skin using 1 % pepsin at various ultrasonic durations (0, 5, 10, 15, and 20 min). The findings indicated that the recovery rate of pepsin-soluble collagen reached its peak at 23.8 % following 15 min of ultrasound treatment. Nonetheless, when the ultrasound action time reached 20 min, the collagen yield was at its lowest [116]. Prolonged ultrasound might lead to the destruction of pepsin and may not sufficiently separate collagen from the skin. According to these findings, it is essential to sustain ultrasonic treatment for the appropriate duration, taking into account the characteristics of the extracted collagen and the ultrasonic method employed.

### 4.2 Environment parameters

#### 4.2.1 Temperature

The temperature of the solution must be maintained at its ideal level. As the temperature increases, molecular kinetic energy rises, facilitating compound diffusion and improving extraction efficiency [117]. The predominant consequence of ultrasounds was the bursting of cavitation bubbles due to the effect of acoustic cavitation. The increase in solution temperature is associated with less intense bubble collapse. Cavitation is reduced at higher extraction temperatures because voids are filled with solvent vapors, leading to less violent collapse; however, at low temperatures, the vapor pressure of solvent is low and allows cavitation bubbles to collapse violently. Changing the temperature of the solution will affect the cavitational intensity, thus affecting the ability of the solvent to penetrate in the cells [118]. Excessive temperatures may compromise the integrity of retrieved collagen. The ambient temperature affects the stability of collagen. Collagen molecules in solution denature at the upper threshold of the physiological temperature or the highest body temperature of the source species. Consequently, deep-sea fish collagen generally exhibits worse thermostability compared to freshwater fish collagen. Mammalian collagens, such as those derived from bovine and swine sources, often exhibit greater denaturation temperatures than fish collagens [119]. Ata et al. investigated the influence of temperature on the extraction of pepsin-soluble collagens from lamb foot using UAE. Their findings indicated the peak collagen concentration at 25 °C across various ultrasonic durations. Collagen extracted at 30 °C exhibited a reduced concentration, apparently owing to structural degradation at elevated temperatures [104]. Consequently, to preserve the integrity of collagen extract’s structure and function, extraction must be performed at an optimal temperature to improve the efficacy of UAE.

#### 4.2.2 Air pressure

Air pressure affects the collapse strength of cavitation bubbles. Higher air pressure increases the cavitation threshold, so stronger ultrasonic energy needs to be used to induce cavitation. Most of the ultrasonic extraction experiments are carried out under atmospheric pressure, with satisfactory outcomes. This indicates that standard air pressure is often sufficient to provide the intended effect of ultrasonic cavitation. Utilizing air pressure facilitates the efficient execution of ultrasonic therapy without requiring further apparatus.

## 5 Ultrasound modification

### 5.1 Effect of US on amino acids profile

The type of extracted collagen is determined by its amino acid profile. The collagen structure is characterized by its amino acid sequence, covalent structure, and post-transcriptional modifications, such as hydroxylation, glycosylation, and cross-linking on the amino acid side chains [120]. Ultrasound-induced cavitation breaks up the complex collagen structure, while the physical field makes the collagen more flexible and improves the interaction between enzymes and substrates. Hydroxyproline and proline represent unique amino acids found within collagen. The collagen extracted from sonicated tuna skin exhibited elevated levels of proline and hydroxyproline in comparison to collagen that had not undergone ultrasonic treatment, with these increases correlating to extended ultrasonic exposure time (0–25 min). Nonetheless, the application of ultrasonic treatment for durations of 10 and 15 min resulted in a reduction of proline and hydroxyproline levels within the collagen, even lower than that without ultrasonic treatment. Ultrasonic treatment promoted the extraction of other components. This leads to dilution effect, which reduces the contents of proline and hydroxyproline in collagen [115]. In summary, the application of ultrasound significantly improves the amino acid profile, elevating levels of proline, hydroxyproline, and free amino acids. Free amino acids are a nutritional component easily digested and absorbed by the human body. They have the function of enhancing the body’s metabolism, growth, and immunity [121]. Additionally, the amino acid composition plays a key role in collagen characteristics, including solubility, structural strength, heat resistance, crosslinking ability, and nutritional value [88]. Generally, higher proline and hydroxyproline content results in obtaining collagen with great structural stability along with higher thermal denaturation [86]. This enhancement elevates both the bioavailability, and functional properties of collagen products, consequently broadening the scope of ultrasound applications in collagen-enriched foods.

### 5.2 Effect of US on the structural modifications of collagen

Understanding the effect of ultrasound on collagen structure is essential since it directly influences the techno-functional qualities of collagen. Ultrasound disrupts insoluble macromolecular clumps by altering the secondary and tertiary structures of proteins. Sonication and cavitation induce sonolysis of polar molecules, resulting in the generation of free radicals and reactive species such as superoxide. These substances induce conformational alterations in proteins’ secondary and tertiary structures by disrupting noncovalent connections and facilitating protein interactions [122].

#### 5.2.1 Secondary structure

**Fig. 4:**
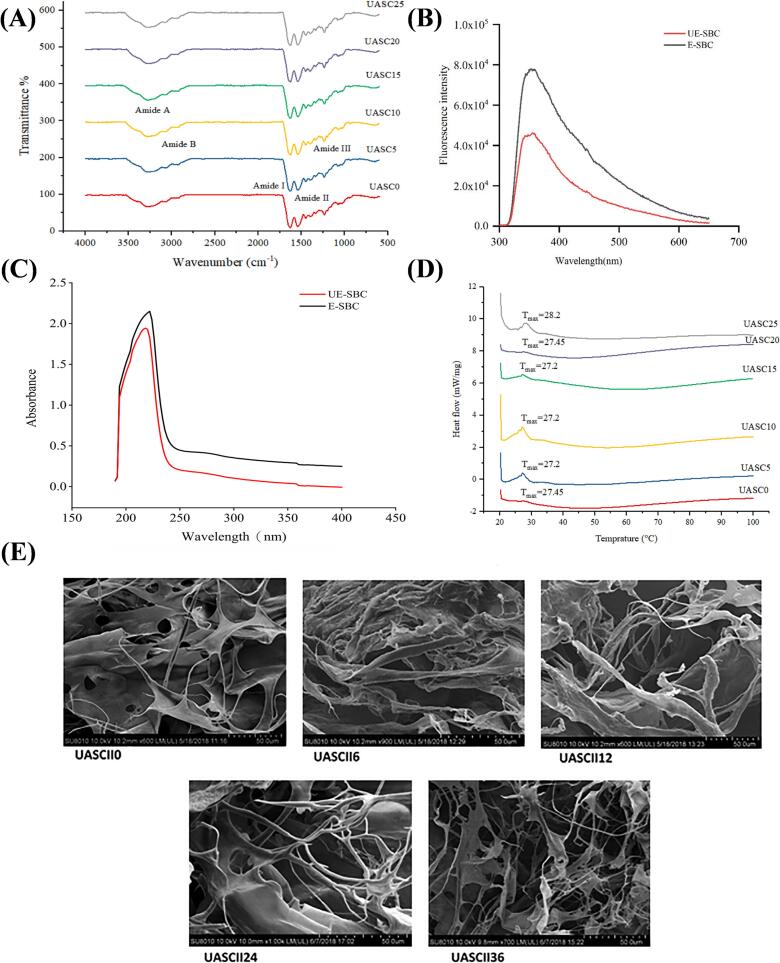
The main methods to evaluate collagen conformational changes. FTIR (A), Fluorescence (B), UV (C) DSC (D), SEM (E).

The modifications in collagen secondary structure are typically evaluated using cutting-edge techniques such as FTIR, CD, and Raman spectroscopy. FTIR is an effective method for identifying alterations in protein secondary structure by the analysis of their infrared radiation absorption. For example, Xu et al. used FTIR to investigate the effect of ultrasound on the structure of collagen hydrolysates derived from deer tendon [123]. Ultrasound treatment led to a decrease in α-helix and β-turn and an increase in β-sheet compared to the non-ultrasound group. Specifically, α-helix and β-turn content decreased, while β-sheet and random coil content increased with longer ultrasonic exposure. Notably, the α-helix content (11.82 % ± 0.51 %) was lowest, and β-sheet content (24.12 % ± 0.43 %) was highest in the 60 min ultrasound group. Intriguingly, the 120 min ultrasound group showed increases in α-helix and β-turn and decreases in β-sheet and random coil compared to the 60-min group. Thus, FTIR spectroscopy demonstrated that ultrasonic treatment altered the secondary structure of collagen and retained the strength of the triple helix structure [86]. Fig. 4(A) shows the FTIR spectra of the tuna collagen samples. It seems that hydrogen bonding interactions appear more pronounced in selected ultrasonically treated collagen samples (USAC-10, USAC-15, USAC-20, and USAC-25) compared to control groups. This phenomenon correlates with the observed redshift in characteristic absorption bands, manifesting as a bathochromic shift to lower wavenumbers in spectroscopic analysis [115]. In addition, the Raman spectroscopy effectively detects protein secondary structure. The CD is also a commonly used optical method for quickly analyzing the secondary structure of protein. For instance, Hu et al derived findings by examining the CD spectroscopy of sheep bone collagen after ultrasonic treatment. The ultrasound-assisted treatment significantly decreased the α-helices content (1.6 %), β-sheets content (21.9 %), and random coils content (28.4 %), while markedly augmenting the β-turns content (51.9 %) [124]. This suggested that the effects of ultrasound on collagens secondary structure depends on many factors such as collagen type, the original aggregation state of the collagens, the degree of collagens denaturation and ultrasound parameters.

#### 5.2.2 Tertiary structure

The tertiary structure of collagen involves the spatial folding and shape of the protein chain. This stable three-dimensional conformation is maintained by non-covalent bonds, which connect the amino acid residues. The tertiary structure of collagen is frequently assessed using fluorescence spectroscopy [125]. As shown in Fig. 4(B), the maximum fluorescence intensity wavelength of sheep bone collagen extracted by ultrasound-assisted enzymatic hydrolysis increased from 353 nm to 355 nm [124]. Following ultrasound-assisted treatment, the fluorescence intensity of sheep collagen notably decreased. Additionally, the side chain structure and microenvironment of proteins are often analyzed by UV. Collagen typically exhibits strong UV characteristic absorption, with absorbance peaks in the range of 210–240 nm [126]. As shown in Fig. 4(C), the sonicated and non-ultrasound treated sheep bone collagen showed maximum absorption peaks at 222 and 218 nm, respectively, which were related to the presence of C-O, –COOH, and CO-NH_2_ in the collagen polypeptide chain [124].

#### 5.2.3 Structural stability

The structural stability of collagen can be evaluated by differential scanning calorimeter (DSC), which can evaluate the thermal properties of protein to understand heat-induced protein denaturation [127]. The DSC spectrum has two endothermic peaks that show the temperature at which collagen denatures (T_d_) and melts (T_m_) when heated. The DSC data indicate that T_m_ and T_d_ significantly increase with longer ultrasound treatment times. Consequently, ultrasound-treated collagen samples exhibit enhanced thermal stability [56]. Fig. 4(D) shows the DSC profiles of collagen samples, the T_m_ values of collagens isolated are ranging from 27.2 up to 28.2 °C for UASC0 and UASC25. Meanwhile, with the more increase in the ultrasonication time from 15 to 25 min, there was an enhancement in thermal stability of collagen samples [115]. Additionally, after 24 min of ultrasonic treatment, the T_m_ of chicken sternal cartilage collagen increased from 62.97 °C to 67.58 °C, and the T_d_ increased from 55.33 °C to 60.86 °C. The changes in the thermal behaviour of collagen after 24 min of ultrasound treatment indicated that Td increased due to the ultrasound waves and cavitation energy that caused a reformation of hydrophobic bonds between the disordered bonds in the protein. Meanwhile, the results suggested the difference in thermal stability of collagen correlated with the physicochemical conversion using ultrasound treatment time during extraction and because of hydration. Ultrasound assistance has been found to damage collagen structure during alkali treatment because the alkali may weaken hydrogen bonds and break parts of covalent bonds in collagen which then facilitates damage by ultrasound [128]. The covalent cross linkages was partially altered by high ultrasound treatment time (36 min), so the T_m_ of UPSCII36 was observed 54.18 °C less than T_m_ of UPSCII24 was 59.17 °C [46]. In addition, according to the report, the T_d_ of general fish collagens is below 30 °C, suggesting that fish collagen is commonly less stable than mammalian collagen [119].

#### 5.2.4 Microstructure

The surface microstructure of collagen is commonly analyzed by scanning electron microscope (SEM), offering crucial insights into sample microstructure, quality, and structural changes. Akram and Zhang observed the microstructure of collagen in chicken sternal cartilage with scanning electron microscopy (Fig. 4(E)). Compared with the microstructure of nonsonicated-treated collagen, the microstructure of sonicated-treated collagen showed numerous porous and loose cross sections with interconnected fiber network pore structures [45]. Similarly, the research conducted by Zou et al. demonstrated that ultrasonic treatment transformed the microstructure of turtle collagen to exhibit a porous configuration resembling a cave, characterized by increased porosity and enlarged pore dimensions [103].

Consequently, ultrasound technology is extensively employed to enhance collagen by modifying its structure for improved functionality. As the diversity of collagen increases, its structure and properties have grown more intricate, necessitating continuous updates in collagen processing techniques. Since numerous effective factors are involved in the sonication of collagens (US parameters, type of collagen, solution concentration, etc.) the US treatment condition must be optimized through well-designed comprehensive experiments in order to obtain appropriate characteristics in the modified collagens. In order to obtain a more comprehensive the impact that various processing methods have on collagen properties, forthcoming research should employ a synergistic approach of effective techniques to evaluate the structural modifications in collagen.

### 5.3 Techno-functional properties of US-assisted collagen

The ultrasound significantly alters collagen, affecting their secondary, tertiary, and quaternary structures, which in turn changes their techno-functional properties. Ultrasound usually improves different techno-functional properties of protein, like its ability to dissolve, its interfacial properties, its rheological properties, and its ability to gel [129,130].

#### 5.3.1 Solubility

**Fig. 5:**
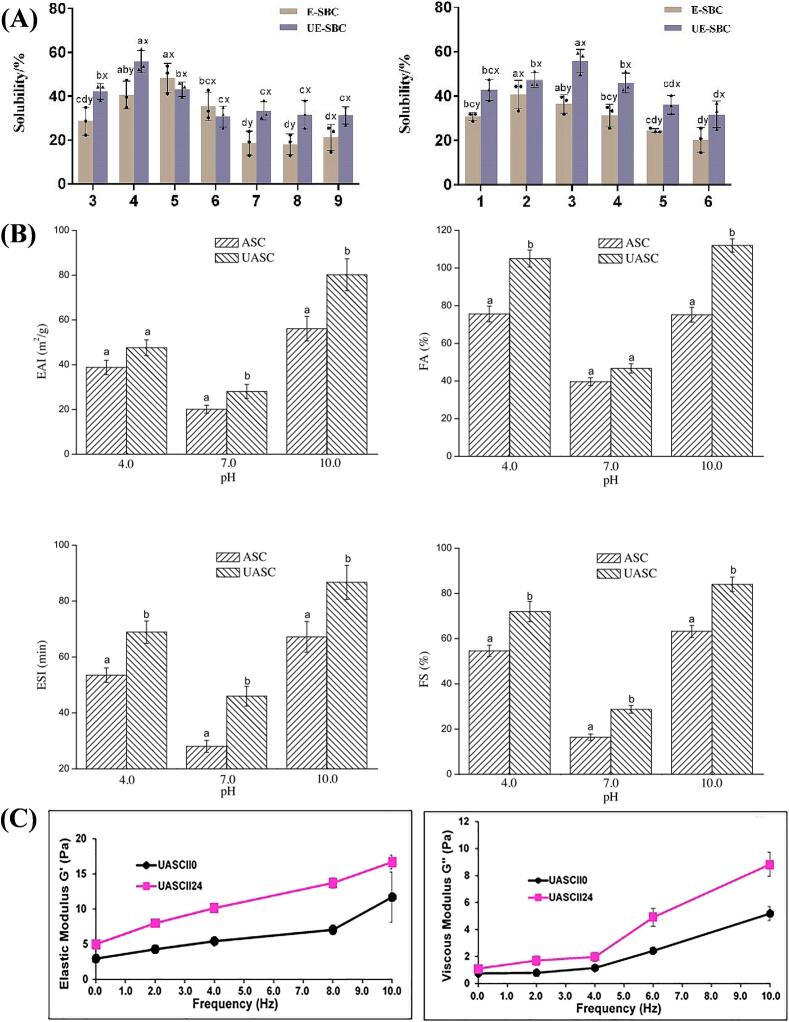
Potential mechanisms of ultrasound to enhance solubility (A), Interfacial properties (B), Rheological properties (C).

In general, protein solubility is mainly affected by environmental factors and protein structure, as well as protein-water interaction and the spatial arrangement of hydrophobic and hydrophilic amino acids on the protein surface. Ultrasound can improve the solubility of collagen in the following ways: (1) The cavitation effect destroys the non-covalent interactions between collagen molecules, such as hydrogen bonding and hydrophobicity, thereby increasing the dispersion and solubility of collagen in solution; (2) ultrasonic vibration energy increases the contact frequency and contact area between solvent molecules and collagen molecules, promotes the interaction between collagen molecules and solvent molecules, and increases solubility; (3) Ultrasonic-induced reaction changes the surface chemical properties, affects the hydrophilicity and hydrophobicity of the collagen surface, and then affects the solubility. As shown in Fig. 5(A), comparing the solubility of sheep bone collagen in saline solutions with different pH and concentrations, it was found that ultrasound-assisted treatment improved the solubility of sheep bone collagen [124]. Similarly, Zou and co-workers documented that ultrasonic treatment improved the solubility of collagen extracted from soft-shelled turtle (*Pelodiscus sinensis*), possibly because the conformation of collagen after ultrasonic treatment changed, which led to increased interaction between hydrophilic amino acid residues and the surrounding solution, thereby enhancing solubility [103].

#### 5.3.2 Interfacial properties

The interfacial properties of proteins are vital for the formation and stability of emulsions and foams. Protein molecules can arrange themselves directly at the oil–water or air–water interface during homogenization. This lowers surface or interfacial tension, which speeds up the formation of bubbles and emulsion droplets [131]. The mechanism of ultrasonic enhancement of collagen interfacial properties involves: (1) The mechanical force of ultrasound promotes the adsorption and desorption of protein molecules at the interface, which contributes to the formation of a more stable protein adsorption layer; (2) Ultrasound-induced collagen molecular chain development; (3) Ultrasonic treatment may promote the formation of a multi-scale structure of collagen at the interface, which is conducive to the stability of emulsion or foam. In this section, we specifically discussed and reviewed the effects of ultrasound on collagen functionality. As shown in Fig. 5(B), the effect of ultrasonic treatment on collagen emulsification properties and foam properties is compared [86]. Furthermore, Hu et al. used ultrasonic-assisted treatment of sheep bone collagen and found that the emulsification and emulsification stability of ultrasonic collagen were generally better than those of non-ultrasonic collagen. In addition, ultrasound-assisted treatment reduced the size of dispersed protein particles, increased the adsorption capacity of oil droplets to proteins, and improved the emulsification performance [124]. Similarly, Akram and Zhang found that ultrasound treatment significantly impacts the emulsification activity and stability of acid-soluble collagen derived from chicken sternal cartilage [45].

#### 5.3.3 Rheological properties

The variation in rheological properties of collagen across different temperatures is a crucial characteristic utilized in various food products and their processing. The elastic modulus (G') represents the elasticity of a protein, whereas the viscous modulus (G“) denotes its viscous behavior [132]. Akram and Zhang studied the impact of ultrasound on chicken sternal cartilage collagen and found that compared with the samples without ultrasound treatment, the samples treated with ultrasound showed a gradual increase as the ultrasonic treatment time and frequency increased (Fig. 5(C)). Compared with the nonsonicated-treated collagen, sonicated-treated collagen G' had a gradual increase trend [45]. For example, in a recent study, authors observed that ultrasonic treatment initially led to a decrease in the elastic modulus (G ') of collagen derived from soft-shelled turtle calipash, followed by an increase, while also significantly diminishing the viscous modulus (G ”). Consequently, ultrasound has the capacity to relax fibrils and bundles, resulting in a less compact and rigid structure, which in turn diminishes G'. Furthermore, ultrasound induces a reduction in the density and thickness of the fibrils within the collagen gel [86]. In conclusion, ultrasonic treatment significantly impacts the rheological properties of collagen.

#### 5.3.4 Water holding and absorption capacity

Water-holding capacity (WHC) refers to the proteins' ability to retain water within their three-dimensional structure, preventing its release or expulsion [46]. The ultrasound-assisted extraction treatment in chicken sternal cartilage, sharpnose stingray (*Dasyatis zugei*) skin, and soft-shelled turtle resulted in higher WHC levels compared to conventional acid extraction [46,71,86]. Water absorption capacity (WAC) is a key functional property of collagen and its products, typically indicating the amount of water absorbed per gram of collagen. Collagen extracted with ultrasound assistance exhibited a water absorption rate of 23 %, outperforming the 18 % rate achieved without ultrasound [86]. Ultrasound improved the water absorption rate of collagen, which was consistent with the result found by Akram and Zhang [45]. The results indicated that ultrasonic-treated collagen contained a higher proportion of hydrophilic groups, which formed hydrogen bonds with water, demonstrating enhanced WHC and WAC capabilities [133].

#### 5.3.5 Oil absorption capacity

The oil absorption capacity (OAC) is recognized as a crucial property of various product ingredients, influencing the flavor and texture of meat and confectionery products [45]. The OAC of collagen was determined using the centrifugation method. The treatment with ultrasound exhibited an increase correlating with extended ultrasonication durations, exceeding the results observed in conventionally treated collagen samples. The research conducted by Akram and Zhang indicates that the OAC value of collagen, measured 24 min post-ultrasound application, was recorded at 11.71 mL/g, surpassing the value of 8.91 mL/g observed in the absence of ultrasound [45]. Furthermore, the observed OAC values exceeded those documented for marine conventional acid-soluble collagen and ultrasound-extracted collagen, which were 6.28 mL/g and 2.57 mL/g, respectively [86]. This could pertain to various factors such as the source of the protein, its classification, the extent of hydrolysis, and the methods employed in processing.

## 6 Preparation of collagen polypeptide by hydrolysis

Collagen peptides, also referred to as hydrolyzed collagen or collagen hydrolysate, are derived from the hydrolysis of native collagen. These peptides typically contain 3–20 amino acids, and their biological activities are largely determined by their amino acid composition [134]. In addition, compared with collagen, low molecular weight collagen hydrolysates demonstrate superior digestibility and bioavailability, along with enhanced biological activity. These hydrolysates also possess improved absorption and processing attributes compared to collagen, making them more readily absorbed and utilized by the human body [135]. In recent years, there has been a growing consumer interest in foods capable of enhancing health or mitigating the risk of disease. Food-derived peptides have garnered significant attention from researchers due to their potential biological activities [136]. Collagen functions as a significant source of biologically active peptides. Currently, the methods employed for the preparation of bioactive peptides primarily encompass synthetic techniques and protein hydrolysis approaches [137]. Given the drawbacks of low yield, numerous by-products, and limited production capacity, the synthetic method is currently unsuitable for large-scale industrial application. Conversely, the approach to acquiring bioactive peptides through proteolysis proves to be more efficient. Consequently, the predominant techniques employed in the preparation of collagen peptides revolve around proteolysis technology, which primarily encompasses chemical hydrolysis, enzymatic hydrolysis, and physical hydrolysis.

### 6.1 Pretreatment procedures

Prior to the hydrolysis of collagen, it is essential to choose appropriate methods based on the various sources of raw materials for collagen extraction, thereby enhancing collagen production [138]. The cross-linked collagen structure in animal tissues exhibits stability, resulting in collagen dissolving at a slow rate, even when subjected to boiling water [120]. Therefore, a mild pretreatment is typically necessary to disrupt the cross-linking among the molecules while preserving the structural integrity of the collagen for extraction purposes [128].

#### 6.1.1 Acid pretreatment

In the process of pretreatment with an acidic solution, the material is immersed in the acidic solution until it penetrates the entire material. At this point, the material will expand to 2 to 3 times its initial volume, and the non-covalent bonds will crack and produce intramolecular interaction bonds [76]. The pretreatment method using an acidic solution is ideal for more delicate raw materials with minimal entanglement of collagen fibers [139].

#### 6.1.2 Alkaline pretreatment

The alkaline pretreatment process involves the application of sodium hydroxide (NaOH) and calcium hydroxide (Ca(OH)_2_), with a duration that may extend from several days to several weeks [76]. This method is appropriate for denser substances, including bovine collagen. Thicker materials typically necessitate a more robust osmotic solution. Nonetheless, the application of NaOH proves to be more efficient in the pretreatment of skin raw materials due to its superior swelling capacity. This results in a more pronounced swelling of the skin raw materials, thereby facilitating collagen extraction by enhancing proton transfer within the tissue matrix [68].

#### 6.1.3 Pretreatment of enzymatic hydrolysis

The pretreatment of raw materials before enzymatic hydrolysis can substantially influence the hydrolysis process by enhancing surface area and partially or fully unfolding the protein, thereby offering more accessible sites for the enzyme [140]. Before enzymatic hydrolysis, various mechanical and chemical pretreatments as well as their combinations, were carried out to increase the yield of the products, protein content, and change the size distribution of the active peptides after enzymatic hydrolysis. At present, the commonly used pretreatment processes include physical methods: high pressure, mechanical crushing, freeze-drying, freeze-grinding, etc., which increase the surface area of raw materials by physical means and improve the contact efficiency of enzymes [141]. Chemical methods: Chemicals such as acids, bases, or surfactants are used to break down the structure of the raw material and make it easier for enzymes to act [128].

### 6.2 Chemical hydrolysis

There are two primary types of chemical methods for collagen hydrolysis: acid hydrolysis and alkaline hydrolysis. The chemical hydrolysis of proteins is considered an effective method for producing peptides and amino acids [142]. However, the disadvantage of chemical hydrolysis is that under harsh conditions, it can partially or completely destroy tryptophan, tyrosine, serine, and threonine. The challenge in chemical hydrolysis of collagen lies in the fact that enhancing acidic or alkaline conditions to reduce molecular weight typically results in increased production of free amino acids. Moreover, both alkaline and acidic processes are highly corrosive to equipment, and the final product after neutralization has a high salt content.

### 6.3 Enzymatic hydrolysis

The food industry extensively employs the process of enzymatic hydrolysis of proteins through the action of proteolytic enzymes. The conditions for enzymatic hydrolysis of proteins are milder than chemical hydrolysis. Enzymatic hydrolysis is the most widely used method for collagen hydrolysis, enhancing the nutritional and functional properties of collagen polypeptides. The molecular bonds between collagen polypeptides are broken down during enzymatic hydrolysis, resulting in the hydrolysis of the polypeptide chains into smaller bioactive peptides with molecular weights ranging from 1 kDa to 4 kDa [142]. The most used commercial enzymes for preparing collagen hydrolysates are papain, neutrase, pepsin, flavourzyme, trypsin, collagenase, and bromelain. The enzymatic hydrolysis process is significantly influenced by critical factors such as temperature, time, pH, and enzyme concentration, which in turn affect peptide generation and the characteristics of the resultant peptide/hydrolysate. The kinetics of enzyme reactions are profoundly influenced by temperature and pH, with these parameters exhibiting variability contingent upon the particular enzyme in question. Careful consideration is essential in enzymatic hydrolysis, particularly regarding the choice of suitable enzymes to attain the intended functional components in protein hydrolysates.

### 6.4 Physical hydrolysis

Physical hydrolysis of collagen peptides mainly refers to physical means that do not involve chemical reagents or biocatalysts (such as enzymes) to achieve the breakdown of collagen. This hydrolysis method usually utilizes physical factors to disrupt the molecular structure of collagen, thereby promoting its hydrolysis. For example, the use of high static water pressure, ohmic heating, pulsed electric field, microwave-assisted extraction, subcritical water hydrolysis, and other physical technologies [143]. At present, the main physical hydrolysis technology commonly used is subcritical water hydrolysis (SWH). The SWH does not require the introduction of other chemicals, does not produce salt and toxic waste, and has the advantage of short reaction times. For example, Park et al. hydrolyzed pig placentas with subcritical water (170 °C, 1 MPa) and detected a minimum molecular weight of 434 Da for collagen hydrolysates [144]. However, in contrast to enzymatic hydrolysis and chemical hydrolysis, SWH incurs the highest energy costs and necessitates the most sophisticated equipment.

### 6.5 Ultrasound-assisted hydrolysis

In recent years, some emerging technologies have been used to assist enzymatic hydrolysis, such as microwave, pulsed electric fields [141], and ultrasound. As a sustainable, clean, and green extraction technology, ultrasound has shown great potential in improving the efficiency and function of collagen extraction as discussed above. Similarly, ultrasound can effectively improve the collagenase hydrolysis effect. Ultrasound can stimulate the hydrolysis of collagen protein in two ways: either by acting as a pretreatment step prior to hydrolysis, or by actively participating in the hydrolysis process and treating it concurrently with enzymatic hydrolysis. For example, Indriani et al. investigated the impacts of ultrasonic-assisted pretreatment and simultaneous treatment on the preparation of Asian bullfrog skin collagen hydrolysate by papain hydrolysis, which could effectively improve the hydrolysis results under different ultrasonic parameters [145]. The study shows the potential of employing ultrasound either directly as a pretreatment method or as a supplementary technique in the extraction process. To improve the ability to extract collagen polypeptides, some studies have focused on the use of ultrasound as a pretreatment method.

An optimal ultrasonic pretreatment duration can enhance collagen peptide production, potentially due to the following factors: (1) Ultrasonic treatment can generate significant turbulence and shear energy within shaped cavitation, leading to structural changes in the protein. This enhancement facilitates the effective binding of the enzyme to the protein substrate and boosts enzymatic efficiency [146]. (2) Ultrasonic treatment loosens the molecular structure of the protein. This exposure of hydrophobic interaction sites increases surface hydrophobicity and results in the release of additional hydrophobic active peptides [123]. It is crucial to note that prolonged ultrasonic treatment will also negatively impact hydrolysis. Ultrasound restructures protein molecules, reduces enzyme binding sites, and lowers the synthesis of active peptides. Extended ultrasonic treatment results in enhanced aggregation of protein molecules, conceals hydrophobic regions, does not create extra free radical reaction sites, and diminishes the synthesis of low molecular weight peptides [123].

The degree of hydrolysis value (DH) is employed to assess the extent of enzymatic proteolysis, as it is closely associated with the functional properties of protein hydrolysates [123]. The hydrolysate prepared by traditional collagenase was used as the control without ultrasonic pretreatment. By eliminating the acid hydrolysis process and shortening the processing time, the process intervention of ultrasonic-assisted extraction was tried. Compared with conventional extraction, both extraction rate and hydrolysis degree increased significantly with ultrasonic pretreatment, ranging from 6.35 % to 16.07 % and DH from 41.9 % to 59.26 % [8]. The free amino acid (FAA) content indicates the hydrolysis process of proteins. The FAA content reflects the degree of proteolysis. The FAA content in the hydrolysate of ultrasonic pretreatment was 0.864 g/100 g, showing a 5.9 % increase, and the FAA content continued to rise with the increase in ultrasonic power [11]. Similarly, the effect of ultrasonic assistance is directly related to the ultrasonic parameters, such as ultrasonic mode (probe type and water bath type), ultrasonic power, action time, and temperature. Lalthanmawii et al. investigated the impact of ultrasonic enzymatic hydrolysis pretreatment on the hydrolyzed products of chicken skin collagen, using an ultrasonic probe, ultrasonic apparatus, and ultrasonic water bath for ultrasonic-assisted extraction. The results indicated that the use of ultrasound significantly enhanced both the yield and the degree of hydrolysis. Among them, the yield of the ultrasonic probe method is 6.21 %, the degree of hydrolysis is 69.90 %, the yield of the ultrasonic bath method is 5.69 %, the degree of hydrolysis is 62.28 % [8]. Xu et al. studied the impact of ultrasonic pretreatment time on collagen hydrolysate from deer tendon. The findings revealed that DH value increased with time and reached the highest value (23.24 ± 0.36 %) at 60 min [123]. Meanwhile, He et al. found that the increase of ultrasonic power could promote the hydrolysis of cowhide gelatin, but the protein recovery rate did not change significantly with the further increase of ultrasonic power [11].

## 7 Biological activities of collagen/collagen peptides

Collagen/collagen peptides have a number of biological activities such as antioxidant, skin health and wound healing, bone health, anti-tumor, hypotensive and hypoglycemic etc. Ultrasonic treatment can not only improve the yield of collagen and collagen peptides, but also improve their biological activity to varying degrees. The effects of ultrasonic treatment on the different biological activities of collagen or collagen peptides are listed in Table 4.

**Table 4:** List of different biological activities of ultrasonic treatment on collagen or collagen peptides.

Sources	Part	Processing parameters	Biological activity	Ref.
Tuna	Tendon	Ultrasonication for 72 h, 50 % amplitude with pulse mode at 10 s of on/off	Antioxidant activity, immunomodulatory activity	[57]
Deer	Tendon	Ultrasonic power of 400 W, temperature of 20℃, treated for 60 min	Antioxidant activities, the proliferation rate of MC3T3-E1 cells	[123]
Asian Bullfrog	Skin	The ultrasonic intensity of 153 W/cm^2^, the single frequency of 20 kHz, 80 % amplitude. 5 s of on/off, temperature is 37–40 °C, 60 min of total time	Antioxidative activities	[145]
Porcine	Bone	Ultrasonic cell disruptor, powers of 450 W and times of 20 min	Antioxidant activity, Anti-inflammatory activity	[147]
Pig	Skin	Ultrasonic cell crusher, with a 0.6 cm flat tip probe at 25 kHz at ultrasonic power 290 W for 40 min (pulse durations of on-time 1 s and off-time 3 s)	ACE-inhibitory activities	[128]
Cattle	Cowhide	Ultrasonic power of 389 W, and times of 25 min	DPP-IV inhibitory activity	[148]

### 7.1 Antioxidant activity

Oxidation is a chemical process that leads to the formation of free radicals, which can potentially harm body cells [149]. Currently, due to certain health concerns, the use of synthetic antioxidants is limited, leading to a growing demand for collagen and collagen peptides as natural antioxidants [150]. Notably, because the peptide is shorter and smaller, it can exert antioxidant activity more efficiently. Compared with the control group, ultrasound-assisted extraction significantly increased the DPPH and ABTS of chicken skin collagen hydrolysate [8]. Under ultrasonic treatment, the antioxidant activity of cow skin gelatin hydrolysate increased from 28 % to 45 % at an ultrasonic power of 300 W. However, at 400 W, the antioxidant activity decreased to about 30 % [11]. Similarly, Xu et al. discovered that the reducing capacity of deer tendon collagen hydrolysate treated with ultrasound (60 min) was 1.81 times that of the non-ultrasonic treatment [123].

### 7.2 Skin health and wound healing

Skin aging significantly affects skin health, causing wrinkles, dryness, sagging, and other adverse effects as people age, leading to the skin losing elasticity and moisture [151]. Research indicates that collagen supplementation can stimulate the proliferation of dermal cells, enhance tissue repair, and thus help maintain skin elasticity, reduce wrinkles, and delay the aging process [135]. Wounds may result from burns, accidents including sports trauma, surgical procedures, traffic injuries, military training injuries, and other unpredictable factors [152]. Collagen deposition plays a crucial role in wound healing. As a key protein in the skin and extracellular matrix (ECM), collagen is a naturally biodegradable macromolecule that facilitates wound repair [153,154]. Due to its superior hemostatic activity and excellent biocompatibility, collagen is often used as a wound dressing in clinical practice [155]. Abdelhedi et al. obtained bioactive peptide by hydrolyzing black striped semi-proboscis gelatin and proved that it has excellent antibacterial activity [156].

### 7.3 Bone health

Collagen constitutes 70 % to 80 % of the organic matter in bone. During bone formation, adequate collagen fibers must be produced initially to establish the bone structure [157]. Osteoblasts are the main components of bone and are essential for the development and formation of bone [158]. Zhu et al. extracted collagen peptide from pig bone and studied its induction effect on osteoblasts. The results showed that low molecular weight peptides were mainly involved in the proliferation and differentiation of MC3T3-E1 [27]. Xu et al. found that the proliferation rate of MC3T3-E1 cells could be improved and the differentiation ability of MC3T3-E1 cells could be better promoted by appropriate ultrasonic pretreatment before collagenic hydrolysis of deer tendon [123]. Arthritis is also a common bone health problem and generally refers to an inflammatory disease that occurs in the joints and surrounding tissues. Collagen also has potential value in treating osteoarthritis and maintaining joint health [159].

### 7.4 Anti-tumor activity

The extracellular matrix (ECM) is crucial in stromal cell interactions, leading to the activation of intracellular signaling pathways, many of which are associated with tumor invasion and metastasis [160]. Shark cartilage is advocated for its potential as an anti-cancer agent, attributed to its anti-tumor properties. The matrix of shark cartilage consists of different types of collagens. Research indicates that the 14 kDa protein derived from shark cartilage has potential applications in dendritic cell-mediated T-cell stimulation and the induction of critical immune responses, including cancer immunotherapy [161].

### 7.5 Hypotensive and hypoglycemic

The angiotensin-I converting enzyme (ACE) is crucial for blood pressure regulation. ACE-inhibiting peptides derived from collagen can be produced from fish and meat waste products [162]. For example, 14 novel ACE-inhibitory peptides have been isolated from tuna hydrolysates [163]. In addition, collagen hydrolysates derived from cattle and pigs exhibit ACE inhibitory activity. Zhang et al. discovered that ultrasonic treatment of hydrolyzed pig skin collagen significantly increased the yield of ACE inhibitory peptides [128].

Type 2 diabetes mellitus (T2DM) is a prevalent metabolic disorder characterized by elevated blood sugar levels, frequently linked to obesity and chronic inflammation [164]. The protein peptide was hydrolyzed from the by-product of squid processing, which has the function of hypoglycemic (DPP-IV inhibition) polypeptide [165].

## 8 Conclusion and future perspectives

This article elucidates the effects and application of ultrasonic technology in the extraction, functionality, and characteristics of collagen. Ultrasound, act as an auxiliary extraction process, significantly enhancing the dissolution of collagen through both the cavitational and mechanical effects. This approach not only increases the yield of collagen extraction, but also preserve the integrity of collagen molecule while enhancing its functional properties. The cavitational effects produced by sonication can induce modifications in the secondary and tertiary structures of collagen to a certain degree, subsequently altering its chemical, biophysical, and surface-active properties. This modification aids in enhancing solubility, interface properties, rheological properties, water retention, and oil absorption. Furthermore, ultrasound can reveal the spatial structure of collagen, providing a beneficial enhancement to traditional modification methods. The application of ultrasound in collagen hydrolysis enhances the biological activity of the hydrolyzed products. Ultrasound effectively enhances traditional hydrolysis methods by disrupting the collagen structure and increasing the availability of hydrolysis sites. Despite the promising outcomes, it is essential to highlight that most studies in this field have predominantly been conducted on a laboratory scale, which limits the commercial viability of ultrasound technology for broader applications.

Due to the abundant sources of collagen, different ultrasonic treatments are required, which have strict requirements for processing conditions. This requires improving the accuracy and functionality of the equipment. Design innovative, efficient, and intelligent ultrasound equipment to promote large scale industrialization and continuously meet the growing market demand for collagen products. Ultimately, combining ultrasound with other innovative technologies to enhance collagen quality through synergistic effects has enormous potential. Future research should focus on exploring the relationship between ultrasound technology and the spatial structure and processing performance of collagen, as well as studying the basic principles of controlling the interaction mechanism between ultrasound and collagen. In addition, further research is needed to evaluate the impact of ultrasound technology on the biological activity of collagen.

## CRediT authorship contribution statement

**Yongjun Zhao:** Writing – review & editing, Writing – original draft, Formal analysis. **Mingming Zhong:** Writing – review & editing, Formal analysis. **Shuixiu Pang:** Supervision, Formal analysis. **Ping Liu:** Supervision, Funding acquisition. **Yufan Sun:** Writing – review & editing, Formal analysis. **Qiufang Liang:** Formal analysis. **Kai Yu:** Formal analysis. **Junxia Wang:** Formal analysis. **Abdur Rehman:** Writing – review & editing, Formal analysis. **Arif Rashid:** Writing – review & editing, Formal analysis. **Haile Ma:** Supervision, Resources. **Xiaofeng Ren:** Writing – review & editing, Funding acquisition.

## Declaration of competing interest

The authors declare that they have no known competing financial interests or personal relationships that could have appeared to influence the work reported in this paper.

## References

[1] M.E. Nimni, R.D. Harkness, Molecular Structure and Functions of Collagen, in: Collagen, CRC Press, 1988.

[2] Subhan F., Hussain Z., Tauseef I., Shehzad A., Wahid F. (2021). A review on recent advances and applications of fish collagen. Crit. Rev. Food Sci. Nutr..

[3] Xu S., Zhao Y., Song W., Zhang C., Wang Q., Li R., Shen Y., Gong S., Li M., Sun L. (2023). Improving the sustainability of processing by-products: extraction and recent biological activities of collagen peptides. Foods.

[4] Oechsle A.M., Akgün D., Krause F., Maier C., Gibis M., Kohlus R., Weiss J. (2016). Microstructure and physical–chemical properties of chicken collagen. Food Struct..

[5] Tang J., Saito T. (2015). Biocompatibility of novel type I collagen purified from tilapia fish scale: an in vitro comparative study. Biomed Res. Int..

[6] Yu Z., Gao Y., Jia X., Cui S., Ma L., Zheng D., Li X., Li L., Zhang L., Chen Y. (2024). Recent advance in high-intensity ultrasound modification of blue food protein: mechanisms, functional properties and structural alterations. Trends Food Sci. Technol..

[7] Larder C.E., Iskandar M.M., Kubow S. (2023). Collagen hydrolysates: a source of bioactive peptides derived from food sources for the treatment of osteoarthritis. Medicines (Basel).

[8] Lalthanmawii J., Banerjee R., Maheswarappa N.B., Biswas S., Belore B., Govindaiah P.M., Patra G. (2024). Ultrasound-assisted extraction for green recovery of poultry skin collagen hydrolysates with antioxidant and antihypertensive activities. Biomass Conv. Bioref..

[9] Kendler S., Kobbenes S.M.M., Jakobsen A.N., Mukhatov K., Lerfall J. (2023). The application of microwave and ultrasound technologies for extracting collagen from European plaice by-products. Front. Sustain. Food Syst..

[10] Li M., Zhou C., Wang B., Zeng S., Mu R., Li G., Li B., Lv W. (2023). Research progress and application of ultrasonic- and microwave-assisted food processing technology. Compr. Rev. Food Sci. Food Saf..

[11] He L., Gao Y., Wang X., Han L., Yu Q., Shi H., Song R. (2021). Ultrasonication promotes extraction of antioxidant peptides from oxhide gelatin by modifying collagen molecule structure. Ultrason. Sonochem..

[12] Yan Q., Li N., Li Y., Zhao Z., Song Q., Lu S., Wang J., Wang Q. (2024). Preparation and identification of novel antioxidant peptides from collagen hydrolysate of sheep hoof assisted by ultrasound. Int. J. Biol. Macromol..

[13] Delikanlı Kıyak B., İnan Çınkır N., Çelebi Y., Durgut Malçok S., Çalışkan Koç G., Adal S., Yüksel A.N., Süfer Ö., Özkan Karabacak A., Ramniwas S., Pandiselvam R. (2024). Advanced technologies for the collagen extraction from food waste – a review on recent progress. Microchemical J..

[14] Shoulders M.D., Raines R.T. (2009). Collagen structure and stability. Annu. Rev. Biochem..

[15] Exposito J.-Y., Valcourt U., Cluzel C., Lethias C. (2010). The Fibrillar collagen family. IJMS.

[16] A. Owczarzy, R. Kurasiński, K. Kulig, W. Rogóż, A. Szkudlarek, M. Maciążek-Jurczyk, Collagen - structure, properties and application, (2020). https://doi.org/10.34821/ENG.BIOMAT.156.2020.17-23.

[17] Han Y., Hu J., Sun G. (2021). Recent advances in skin collagen: functionality and non-medical applications. J. Leather Sci. Eng..

[18] Jin W.-G., Pei J., Du Y.-N., Pan J., Gao R., Chen D.-J., Wu H.-T., Zhu B.-W. (2019). Characterization and functional properties of Gelatin extracted from Chinese giant salamander (*Andrias Davidianus*) skin. J. Aquat. Food Prod. Technol..

[19] O’Leary L.E.R., Fallas J.A., Bakota E.L., Kang M.K., Hartgerink J.D. (2011). Multi-hierarchical self-assembly of a collagen mimetic peptide from triple helix to nanofibre and hydrogel. Nat. Chem..

[20] Myllyharju J., Lamberg A., Notbohm H., Fietzek P.P., Pihlajaniemi T., Kivirikko K.I. (1997). Expression of wild-type and modified proα chains of human type I procollagen in insect cells leads to the formation of stable [α1(I)]2α2(I) Collagen heterotrimers and [α1(I)]3 homotrimers but not [α2(I)]3 homotrimers*. J. Biol. Chem..

[21] Xu X., Gan Q., Clough R.C., Pappu K.M., Howard J.A., Baez J.A., Wang K. (2011). Hydroxylation of recombinant human collagen type I alpha 1 in transgenic maize co-expressed with a recombinant human prolyl 4-hydroxylase. BMC Biotechnol..

[22] Ferraro V., Gaillard-Martinie B., Sayd T., Chambon C., Anton M., Santé-Lhoutellier V. (2017). Collagen type I from bovine bone. Effect of animal age, bone anatomy and drying methodology on extraction yield, self-assembly, thermal behaviour and electrokinetic potential. Int. J. Biol. Macromol..

[23] Silvipriya K., Kumar K., Bhat A., Kumar B., John A., Lakshmanan P. (2015). Collagen: animal sources and biomedical application. J. Appl. Pharm. Sci..

[24] Rizk M.A., Mostafa N.Y. (2016). Extraction and characterization of collagen from buffalo skin for biomedical applications. Orient. J. Chem..

[25] Wang H. (2021). A review of the effects of collagen treatment in clinical studies. Polymers.

[26] Choi D., Min S.-G., Jo Y.-J. (2018). Functionality of porcine skin hydrolysates produced by hydrothermal processing for liposomal delivery system. J. Food Biochem..

[27] Zhu L., Xie Y., Wen B., Ye M., Liu Y., Imam K.M.S.U., Cai H., Zhang C., Wang F., Xin F. (2020). Porcine bone collagen peptides promote osteoblast proliferation and differentiation by activating the PI3K/Akt signaling pathway. J. Funct. Foods.

[28] Wen-Chien L., Chien-Shan C., Yung-Jia C., Tresiliana M.A., Po-Hsien L. (2023). Characterization and biological properties of marine by-product collagen through ultrasound-assisted extraction. Aquacult. Rep..

[29] Oslan S.N.H., Li C.X., Shapawi R., Mokhtar R.A.M., Noordin W.N.M., Huda N. (2022). Extraction and characterization of bioactive fish by-product collagen as promising for potential wound healing agent in pharmaceutical applications: current trend and future perspective. Int. J. Food Sci..

[30] Laasri I., Bakkali M., Mejias L., Laglaoui A. (2023). Marine collagen: unveiling the blue resource-extraction techniques and multifaceted applications. Int. J. Biol. Macromol..

[31] Ahmed R., Haq M., Chun B.-S. (2019). Characterization of marine derived collagen extracted from the by-products of bigeye tuna (Thunnus obesus). Int. J. Biol. Macromol..

[32] Kitsanapong K., Ali H., Jirawat Y. (2023). Ultrasound-assisted extraction of collagen from broiler chicken trachea and its biochemical characterization. Ultrason. Sonochem..

[33] Zou Y., Li L., Yang J., Yang B., Ma J., Wang D., Xu W. (2022). Effect of ultrasound assisted collagen peptide of chicken cartilage on storage quality of chicken breast meat. Ultrason. Sonochem..

[34] Pei Y., Yang W., Tang K., Kaplan D.L. (2023). Collagen processing with mesoscale aggregates as templates and building blocks. Biotechnol. Adv..

[35] Naomi R., Ridzuan P.M., Bahari H. (2021). Current insights into collagen type I. Polymers.

[36] Samad N., Sikarwar A. (2016). Collagen: new dimension in cosmetic and healthcare. IJBCRR.

[37] Liu D., Nikoo M., Boran G., Zhou P., Regenstein J.M. (2015). Collagen and gelatin. Annu. Rev. Food Sci. Technol..

[38] Gómez-Guillén M.C., Giménez B., López-Caballero M.E., Montero M.P. (2011). Functional and bioactive properties of collagen and gelatin from alternative sources: a review. Food Hydrocoll..

[39] Karayannakidis P.D., Zotos A. (2016). Fish processing by-products as a potential source of gelatin: a review. J. Aquat. Food Prod. Technol..

[40] Avila Rodríguez M.I., Rodríguez Barroso L.G., Sánchez M.L. (2018). Collagen: a review on its sources and potential cosmetic applications. J. Cosmet. Dermatol..

[41] Jeevithan E., Bao B., Bu Y., Zhou Y., Zhao Q., Wu W. (2014). Type II collagen and gelatin from silvertip shark (Carcharhinus albimarginatus) cartilage: isolation, purification, physicochemical and antioxidant properties. Mar. Drugs.

[42] Furtado M., Chen L., Chen Z., Chen A., Cui W. (2022). Development of fish collagen in tissue regeneration and drug delivery. Eng. Regener..

[43] Kim H.-W., Yeo I.-J., Hwang K.-E., Song D.-H., Kim Y.-J., Ham Y.-K., Jeong T.-J., Choi Y.-S., Kim C.-J. (2016). Isolation and characterization of pepsin-soluble collagens from bones, skins, and tendons in duck feet. Korean J. Food Sci. Anim. Resour..

[44] Xin Du., Haijing L.i., Maheshati N., Shuo S., Baohua K., Qian L., Xiufang X. (2021). Application of ultrasound treatment in chicken gizzards tenderization: effects on muscle fiber and connective tissue. Ultrason. Sonochem..

[45] Akram A.N., Zhang C. (2020). Effect of ultrasonication on the yield, functional and physicochemical characteristics of collagen-II from chicken sternal cartilage. Food Chem..

[46] Akram A.N., Zhang C. (2020). Extraction of collagen-II with pepsin and ultrasound treatment from chicken sternal cartilage; physicochemical and functional properties. Ultrason. Sonochem..

[47] Jayaprakash S., Mohamad Abdul Razeen Z., Naveen Kumar R., He J., Milky M.G., Renuka R., Sanskrithi M.V. (2024). Enriched characteristics of poultry collagen over other sources of collagen and its extraction methods: a review. Int. J. Biol. Macromol..

[48] Wang T., Lew J., Premkumar J., Poh C.L., Win Naing M. (2017). Production of recombinant collagen: state of the art and challenges. Eng. Biol..

[49] Sorushanova A., Delgado L.M., Wu Z., Shologu N., Kshirsagar A., Raghunath R., Mullen A.M., Bayon Y., Pandit A., Raghunath M., Zeugolis D.I. (2019). The Collagen suprafamily: from biosynthesis to advanced biomaterial development. Adv. Mater..

[50] Guo X., Ma Y., Wang H., Yin H., Shi X., Chen Y., Gao G., Sun L., Wang J., Wang Y., Fan D. (2024). Status and developmental trends in recombinant collagen preparation technology. Regen Biomater..

[51] Zhao Z., Deng J., Fan D. (2023). Green biomanufacturing in recombinant collagen biosynthesis: trends and selection in various expression systems. Biomater. Sci..

[52] Wang L., Li P., Ren Y., Bai F., Wang J., Zhang Y., Jin W., El-Seedi H., Gao R. (2021). A novel extraction approach and unique physicochemical properties of gelatin from the swim bladder of sturgeon. J. Sci. Food Agric..

[53] Guo Y., Li X., Jia W., Huang F., Liu Y., Zhang C. (2021). Characterization of an intracellular aspartic protease (PsAPA) from Penicillium sp. XT7 and its application in collagen extraction. Food Chem..

[54] Noorzai S., Verbeek C.J.R., Lay M.C., Swan J. (2020). Collagen extraction from various waste bovine hide sources. Waste Biomass Valor..

[55] Ong T.Y., Shaik M.I., Sarbon N.M. (2021). Isolation and characterization of acid and pepsin soluble collagen extracted from sharpnose stingray (Dasyatis zugei) skin. Food Res..

[56] Ali A.M.M., Kishimura H., Benjakul S. (2018). Extraction efficiency and characteristics of acid and pepsin soluble collagens from the skin of golden carp (Probarbus Jullieni) as affected by ultrasonication. Process Biochem..

[57] Chanmangkang S., Maneerote J., Surayot U., Panya A., You S., Wangtueai S. (2024). Physicochemical and biological properties of collagens obtained from tuna tendon by using the ultrasound-assisted extraction. J. Agric. Food Res..

[58] Asaduzzaman A.K.M., Getachew A.T., Cho Y.-J., Park J.-S., Haq M., Chun B.-S. (2020). Characterization of pepsin-solubilised collagen recovered from mackerel (*Scomber japonicus*) bone and skin using subcritical water hydrolysis. Int. J. Biol. Macromol..

[59] Blanco M., Vázquez J.A., Pérez-Martín R.I., Sotelo C.G. (2019). Collagen extraction optimization from the skin of the small-spotted Catshark (S. canicula) by response surface methodology. Mar. Drugs.

[60] Cao C., Wang H., Zhang J., Kan H., Liu Y., Guo L., Tong H., Wu Y., Ge C. (2023). Effects of extraction methods on the characteristics, physicochemical properties and sensory quality of collagen from spent-hens bones. Foods.

[61] Silva I., Vaz B.M.C., Sousa S., Pintado M.M., Coscueta E.R., Ventura S.P.M. (2024). Gastrointestinal delivery of codfish skin-derived collagen hydrolysates: deep eutectic solvent extraction and bioactivity analysis. Food Res. Int..

[62] Dhakal D., Koomsap P., Lamichhane A., Sadiq M.B., Anal A.K. (2018). Optimization of collagen extraction from chicken feet by papain hydrolysis and synthesis of chicken feet collagen based biopolymeric fibres. Food Biosci..

[63] Sousa R.O., Martins E., Carvalho D.N., Alves A.L., Oliveira C., Duarte A.R.C., Silva T.H., Reis R.L. (2020). Collagen from Atlantic cod (Gadus morhua) skins extracted using CO_2_ acidified water with potential application in healthcare. J. Polym. Res..

[64] Ampitiya A.G.D.M., Gonapinuwala S.T., Fernando C.A.N., De Croos M.D.S.T. (2023). Extraction and characterisation of type I collagen from the skin offcuts generated at the commercial fish processing centres. J. Food Sci. Technol..

[65] Matinong A.M.E., Chisti Y., Pickering K.L., Haverkamp R.G. (2022). Collagen extraction from animal skin. Biology.

[66] Prajaputra V., Isnaini N., Maryam S., Ernawati E., Deliana F., Haridhi H.A., Fadli N., Karina S., Agustina S., Nurfadillah N., Arisa I.I., Desiyana L.S., Bakri T.K. (2024). Exploring marine collagen: sustainable sourcing, extraction methods, and cosmetic applications. S. Afr. J. Chem. Eng..

[67] M.M. Schmidt, R. Prestes Dornelles, R. Mello, E.H. Kubota, M. Mazutti, A. Kempka, I. Demiate, Collagen extraction process, 23 (2016) 913–922.

[68] Liu D., Wei G., Li T., Hu J., Lu N., Regenstein J.M., Zhou P. (2015). Effects of alkaline pretreatments and acid extraction conditions on the acid-soluble collagen from grass carp (*Ctenopharyngodon idella*) skin. Food Chem..

[69] Zhu L., Li J., Wang Y., Sun X., Li B., Poungchawanwong S., Hou H. (2020). Structural feature and self-assembly properties of type II collagens from the cartilages of skate and sturgeon. Food Chem..

[70] Seixas M.J., Martins E., Reis R.L., Silva T.H. (2020). Extraction and characterization of collagen from elasmobranch byproducts for potential biomaterial use. Mar. Drugs.

[71] Shaik M.I., Asrul Effendi N.F., Sarbon N.M. (2021). Functional properties of sharpnose stingray (*Dasyatis zugei*) skin collagen by ultrasonication extraction as influenced by organic and inorganic acids. Biocatal. Agric. Biotechnol..

[72] Li K., Ma H., Li S., Zhang C., Dai C. (2017). Effect of ultrasound on alkali extraction protein from rice dreg flour. J. Food Process Eng..

[73] Zhang Z., Wang Y., Dai C., He R., Ma H. (2018). Alkali extraction of rice residue protein isolates: effects of alkali treatment conditions on lysinoalanine formation and structural characterization of lysinoalanine-containing protein. Food Chem..

[74] Petcharat T., Benjakul S., Karnjanapratum S., Nalinanon S. (2021). Ultrasound-assisted extraction of collagen from clown featherback (Chitala ornata) skin: yield and molecular characteristics. J. Sci. Food Agric..

[75] Liang Q., Wang L., Sun W., Wang Z., Xu J., Ma H. (2014). Isolation and characterization of collagen from the cartilage of Amur sturgeon (*Acipenser schrenckii*). Process Biochem..

[76] Jafari H., Lista A., Siekapen M.M., Ghaffari-Bohlouli P., Nie L., Alimoradi H., Shavandi A. (2020). Fish collagen: extraction, characterization, and applications for biomaterials engineering. Polymers.

[77] Zhang J., Wen C., Zhang H., Duan Y., Ma H. (2020). Recent advances in the extraction of bioactive compounds with subcritical water: a review. Trends Food Sci. Technol..

[78] Zhou C., Okonkwo C.E., Inyinbor A.A., Yagoub A.E.A., Olaniran A.F. (2023). Ultrasound, infrared and its assisted technology, a promising tool in physical food processing: a review of recent developments. Crit. Rev. Food Sci. Nutr..

[79] Shen L., Pang S., Zhong M., Sun Y., Qayum A., Liu Y., Rashid A., Xu B., Liang Q., Ma H., Ren X. (2023). A comprehensive review of ultrasonic assisted extraction (UAE) for bioactive components: principles, advantages, equipment, and combined technologies. Ultrason. Sonochem..

[80] Wang X., Majzoobi M., Farahnaky A. (2020). Ultrasound-assisted modification of functional properties and biological activity of biopolymers: a review. Ultrason. Sonochem..

[81] Yarley O.P.N., Jiang H., Zhou C., Yang H. (2018). *Sorghum Bicolor* L. leaf sheath polysaccharides: dual frequency ultrasound-assisted extraction and desalination. Ind. Crop Prod..

[82] Mtetwa M.D., Qian L., Zhu H., Cui F., Zan X., Sun W., Wu D., Yang Y. (2020). Ultrasound-assisted extraction and antioxidant activity of polysaccharides from *Acanthus ilicifolius*. J. Food Meas. Charact..

[83] Li D., Mu C., Cai S., Lin W. (2009). Ultrasonic irradiation in the enzymatic extraction of collagen. Ultrason. Sonochem..

[84] Senadheera T.R.L., Dave D., Shahidi F. (2020). Sea cucumber derived type I collagen: a comprehensive review. Mar. Drugs.

[85] Musa A., Gasmalla M.A.A., Ma H., Sarpong F., Wali A., Awad F.N., Duan Y. (2019). Effect of a multi-frequency counter-current S-type ultrasound pretreatment on the defatted corn germ protein: enzymatic hydrolysis, ACE inhibitory activity and structural characterization. Food Funct..

[86] Zou Y., Wang L., Cai P., Li P., Zhang M., Sun Z., Sun C., Xu W., Wang D. (2017). Effect of ultrasound assisted extraction on the physicochemical and functional properties of collagen from soft-shelled turtle calipash. Int. J. Biol. Macromol..

[87] Ran X.-G., Wang L.-Y. (2014). Use of ultrasonic and pepsin treatment in tandem for collagen extraction from meat industry by-products: ultrasound and pepsin to extract collagen from meat by-products. J. Sci. Food Agric..

[88] Indriani S., Benjakul S., Quan T.H., Sitanggang A.B., Chaijan M., Kaewthong P., Petcharat T., Karnjanapratum S. (2023). Effect of different ultrasound-assisted process modes on extraction yield and molecular characteristics of pepsin-soluble collagen from asian bullfrog skin. Food Bioproc. Tech..

[89] Jin H.-X., Xu H.-P., Li Y., Zhang Q.-W., Xie H. (2019). Preparation and evaluation of peptides with potential antioxidant activity by microwave assisted enzymatic hydrolysis of collagen from sea cucumber acaudina molpadioides obtained from Zhejiang Province in China. Mar. Drugs.

[90] Lin Y.-J., Le G.-W., Wang J.-Y., Li Y.-X., Shi Y.-H., Sun J. (2010). Antioxidative peptides derived from enzyme hydrolysis of bone collagen after microwave assisted acid pre-treatment and nitrogen protection. Int. J. Mol. Sci..

[91] Cui Q., Liu J.-Z., Wang L.-T., Kang Y.-F., Meng Y., Jiao J., Fu Y.-J. (2018). Sustainable deep eutectic solvents preparation and their efficiency in extraction and enrichment of main bioactive flavonoids from sea buckthorn leaves. J. Clean. Prod..

[92] Xu Y., Jiao Y., Luo J., He Z., Zeng M., Shen Q., Chen J., Quan W. (2022). The Influence of deep eutectic solvents extract from ginger on the formation of heterocyclic amines and advanced glycation end products in roast beef patties. Foods.

[93] Tsvetov N., Paukshta O., Fokina N., Volodina N., Samarov A. (2023). Application of natural deep eutectic solvents for extraction of bioactive components from *Rhodiola rosea* (L.). Molecules.

[94] Batista M.P., Fernández N., Gaspar F.B., Bronze M.do R., Duarte A.R.C. (2022). Extraction of biocompatible collagen from blue shark skins through the conventional extraction process intensification using natural deep eutectic solvents. Front. Chem..

[95] Mikšovsky P., Kornpointner C., Parandeh Z., Goessinger M., Bica-Schröder K., Halbwirth H. (2024). Enzyme-assisted supercritical fluid extraction of flavonoids from apple pomace (Malus×domestica). ChemSusChem.

[96] Herzyk F., Piłakowska-Pietras D., Korzeniowska M. (2024). Supercritical extraction techniques for obtaining biologically active substances from a variety of plant byproducts. Foods.

[97] Phon S., Pradana A.L., Thanasupsin S.P. (2023). Recovery of collagen/gelatin from fish waste with carbon dioxide as a green solvent: an optimization and characterization. Recycling.

[98] Luo L., Yang Z., Wang H., Ashokkumar M., Hemar Y. (2022). Impacts of sonication and high hydrostatic pressure on the structural and physicochemical properties of quinoa protein isolate dispersions at acidic, neutral and alkaline pHs. Ultrason. Sonochem..

[99] Yan J.-K., Wang C., Qiu W.-Y., Chen T.-T., Yang Y., Wang W.-H., Zhang H.-N. (2021). Ultrasonic treatment at different pH values affects the macromolecular, structural, and rheological characteristics of citrus pectin. Food Chem..

[100] Bhargava N., Mor R.S., Kumar K., Sharanagat V.S. (2021). Advances in application of ultrasound in food processing: a review. Ultrason. Sonochem..

[101] Bajerová P., Adam M., Bajer T., Ventura K. (2014). Comparison of various techniques for the extraction and determination of antioxidants in plants. J. Sep. Sci..

[102] Adamou P., Harkou E., Villa A., Constantinou A., Dimitratos N. (2024). Ultrasonic reactor set-ups and applications: a review. Ultrason. Sonochem..

[103] Zou Y., Xu P., Li P., Cai P., Zhang M., Sun Z., Sun C., Xu W., Wang D. (2017). Effect of ultrasound pre-treatment on the characterization and properties of collagen extracted from soft-shelled turtle (*Pelodiscus sinensis*). LWT Food Sci. Technol..

[104] Ata O., Kumcuoglu S., Tavman S. (2022). Effects of sonication on the extraction of pepsin-soluble collagens from lamb feet and product characterization. LWT.

[105] Ata O., Bakar B., Turkoz B.K., Kumcuoglu S., Aydogdu Y., Gumustas B., Doganay G.D., Basturk E., Tavman S. (2024). Structural and molecular characterization of collagen-type I extracted from lamb feet. J. Food Sci..

[106] Carreira-Casais A., Otero P., Garcia-Perez P., Garcia-Oliveira P., Pereira A.G., Carpena M., Soria-Lopez A., Simal-Gandara J., Prieto M.A. (2021). Benefits and drawbacks of ultrasound-assisted extraction for the recovery of bioactive compounds from marine algae. Int. J. Environ. Res. Public Health.

[107] Kim H.K., Kim Y.H., Kim Y.J., Park H.J., Lee N.H. (2012). Effects of ultrasonic treatment on collagen extraction from skins of the sea bass *Lateolabrax japonicus*. Fish. Sci..

[108] Wang X., Zhang L., Chen L., Wang Y., Okonkwo C.E., Yagoub A.-E.-G.-A., Wahia H., Zhou C. (2023). Application of ultrasound and its real-time monitoring of the acoustic field during processing of tofu: parameter optimization, protein modification, and potential mechanism. Compr. Rev. Food Sci. Food Saf..

[109] D.N. V, A. Francescutto, C. P, S. F, Temperature Dependence of Cavitation Activity at Different Ultrasound Intensity, (2005). http://hdl.handle.net/11368/2559311 (accessed June 20, 2025).

[110] Kobus Z., Krzywicka M., Pecyna A., Buczaj A. (2021). Process efficiency and energy consumption during the ultrasound-assisted extraction of bioactive substances from hawthorn berries. Energies.

[111] Chen Z.-L., Wang C., Ma H., Ma Y., Yan J.-K. (2021). Physicochemical and functional characteristics of polysaccharides from okra extracted by using ultrasound at different frequencies. Food Chem..

[112] Jun S., Mu Y., Hui J., Obadi M., Chen Z., Bin X. (2020). Effects of single- and dual-frequency ultrasound on the functionality of egg white protein. J. Food Eng..

[113] Zheng X., Zou B., Zhang J., Cai W., Na X., Du M., Zhu B., Wu C. (2024). Recent advances of ultrasound-assisted technology on aquatic protein processing: extraction, modification, and freezing/thawing-induced oxidation. Trends Food Sci. Technol..

[114] Cheng Y., Shi X., Yeboah G.B., Chen L., Wu J. (2024). Effect of multi-mode divergent ultrasound pretreatment on hardness, microstructure and digestion of acid-induced whey protein gels. Foods.

[115] Pezeshk S., Rezaei M., Abdollahi M. (2022). Impact of ultrasound on extractability of native collagen from tuna by-product and its ultrastructure and physicochemical attributes. Ultrason. Sonochem..

[116] Heidari M.G., Rezaei M. (2022). Extracted pepsin of trout waste and ultrasound-promoted method for green recovery of fish collagen. Sustain. Chem. Pharm..

[117] Tchabo W., Ma Y., Kwaw E., Xiao L., Wu M., Apaliya M.T. (2018). Impact of extraction parameters and their optimization on the nutraceuticals and antioxidant properties of aqueous extract mulberry leaf. Int. J. Food Prop..

[118] Panda D., Manickam S. (2019). Cavitation technology—the future of greener extraction method: a review on the extraction of natural products and process intensification mechanism and perspectives. Appl. Sci..

[119] Sionkowska A., Kozłowska J., Skorupska M., Michalska M. (2015). Isolation and characterization of collagen from the skin of *Brama australis*. Int. J. Biol. Macromol..

[120] Hong H., Fan H., Chalamaiah M., Wu J. (2019). Preparation of low-molecular-weight, collagen hydrolysates (peptides): current progress, challenges, and future perspectives. Food Chem..

[121] Rozi P., Mattohti W., Ababakri G., Pengfei L., Yanping C., Yuan L. (2024). Isolation, structure identification, and antioxidant activity of collagen peptides from horse bone marrow. Food Measure.

[122] Mao C., Wu J., Zhang X., Ma F., Cheng Y. (2020). Improving the solubility and digestibility of potato protein with an online ultrasound-assisted PH shifting treatment at medium temperature. Foods.

[123] Xu X., Wang D., Li J., Zeng X., Zhang Z., Zhu J., Liu G., Zhang J., Liang L., Liu X., Li Y., Wen C. (2023). Collagen hydrolysates from deer tendon: Preparation assisted with different ultrasound pretreatment times and promotion in MC3T3-E1 cell proliferation and antioxidant activities. Process Biochem..

[124] Hu G., Li X., Su R., Corazzin M., Liu X., Dou L., Sun L., Zhao L., Su L., Tian J., Jin Y. (2023). Effects of ultrasound on the structural and functional properties of sheep bone collagen. Ultrason. Sonochem..

[125] Cai L., Zhang W., Cao A., Cao M., Li J. (2019). Effects of ultrasonics combined with far infrared or microwave thawing on protein denaturation and moisture migration of *Sciaenops ocellatus* (red drum). Ultrason. Sonochem..

[126] Kozlowska J., Sionkowska A., Skopinska-Wisniewska J., Piechowicz K. (2015). Northern pike (*Esox lucius*) collagen: extraction, characterization and potential application. Int. J. Biol. Macromol..

[127] Kaushik P., Dowling K., McKnight S., Barrow C.J., Wang B., Adhikari B. (2016). Preparation, characterization and functional properties of flax seed protein isolate. Food Chem..

[128] Zhang Y., Ma L., Cai L., Liu Y., Li J. (2017). Effect of combined ultrasonic and alkali pretreatment on enzymatic preparation of angiotensin converting enzyme (ACE) inhibitory peptides from native collagenous materials. Ultrason. Sonochem..

[129] Wang H., Yang H., Chen X., Shen Q. (2022). Structural basis for high-intensity ultrasound treatment in the rheology of myofibrillar protein extracted from *White Croaker* in relation to their solubility. LWT.

[130] Deng X., Ni X., Han J., Yao W., Fang Y., Zhu Q., Xu M. (2023). High-intensity ultrasound modified the functional properties of *Neosalanx taihuensis* myofibrillar protein and improved its emulsion stability. Ultrason. Sonochem..

[131] Shi T., Liu H., Song T., Xiong Z., Yuan L., McClements D.J., Jin W., Sun Q., Gao R. (2021). Use of L-arginine-assisted ultrasonic treatment to change the molecular and interfacial characteristics of fish myosin and enhance the physical stability of the emulsion. Food Chem..

[132] Mu Y., Sun J., Obadi M., Chen Z., Xu B. (2020). Effects of saccharides on the rheological and gelling properties and water mobility of egg white protein. Food Hydrocoll..

[133] Hou H., Li B., Zhang Z., Xue C., Yu G., Wang J., Bao Y., Bu L., Sun J., Peng Z., Su S. (2012). Moisture absorption and retention properties, and activity in alleviating skin photodamage of collagen polypeptide from marine fish skin. Food Chem..

[134] Ahmed M., Verma A.K., Patel R. (2020). Collagen extraction and recent biological activities of collagen peptides derived from sea-food waste: a review. Sustain. Chem. Pharm..

[135] Wang L., Zhang Y., Zhu Z., Zheng F., Gao R. (2023). Food-derived collagen peptides: safety, metabolism, and anti-skin-aging effects. Curr. Opin. Food Sci..

[136] Zhu D., Yuan Z., Wu D., Wu C., El-Seedi H.R., Du M. (2023). The dual-function of bioactive peptides derived from oyster *(Crassostrea gigas)* proteins hydrolysates. Food Sci. Human Wellness.

[137] Raja K., Suresh K., Anbalagan S., Ragini Y.P., Kadirvel V. (2024). Investigating the nutritional viability of marine-derived protein for sustainable future development. Food Chem..

[138] León-López A., Morales-Peñaloza A., Martínez-Juárez V.M., Vargas-Torres A., Zeugolis D.I., Aguirre-Álvarez G. (2019). Hydrolyzed collagen—sources and applications. Molecules.

[139] Hong H., Roy B.C., Chalamaiah M., Bruce H.L., Wu J. (2018). Pretreatment with formic acid enhances the production of small peptides from highly cross-linked collagen of spent hens. Food Chem..

[140] Jin J., Ma H., Wang W., Luo M., Wang B., Qu W., He R., Owusu J., Li Y. (2016). Effects and mechanism of ultrasound pretreatment on rapeseed protein enzymolysis. J. Sci. Food Agric..

[141] Lian K., Maribu I., Rode T.M., Jenssen M., Vang B., Solstad R.G. (2024). More sustainable use of aquaculture cleaner fish: collagen-rich protein hydrolysates from lumpfish (*Cyclopterus lumpus*) – effects of biomass, pretreatment, and enzyme choice. Front. Sustain. Food Syst..

[142] Pal G.K., Suresh P.V. (2016). Sustainable valorisation of seafood by-products: recovery of collagen and development of collagen-based novel functional food ingredients. Innov. Food Sci. Emerg. Technol..

[143] Ulug S.K., Jahandideh F., Wu J. (2021). Novel technologies for the production of bioactive peptides. Trends Food Sci. Technol..

[144] Park S.H., Kim J.-H., Min S.-G., Jo Y.-J., Chun J.-Y. (2015). Effects of ethanol addition on the efficiency of subcritical water extraction of proteins and amino acids from porcine placenta. Korean J. Food Sci. Anim. Resour..

[145] Indriani S., Sae-leaw T., Benjakul S., Hong Quan T., Karnjanapratum S., Nalinanon S. (2022). Impact of different ultrasound-assisted processes for preparation of collagen hydrolysates from Asian bullfrog skin on characteristics and antioxidative properties. Ultrason. Sonochem..

[146] Wen C., Zhang J., Zhou J., Feng Y., Duan Y., Zhang H., Ma H. (2020). Slit divergent ultrasound pretreatment assisted watermelon seed protein enzymolysis and the antioxidant activity of its hydrolysates in vitro and in vivo. Food Chem..

[147] Hao Y., Xing L., Wang Z., Cai J., Toldrá F., Zhang W. (2023). Study on the anti-inflammatory activity of the porcine bone collagen peptides prepared by ultrasound-assisted enzymatic hydrolysis. Ultrason. Sonochem..

[148] He L., Wang X., Wang Y., Luo J., Zhao Y., Han G., Han L., Yu Q. (2023). Production and identification of dipeptidyl peptidase IV (DPP-IV) inhibitory peptides from discarded cowhide collagen. Food Chem..

[149] Xu B., Dong Q., Yu C., Chen H., Zhao Y., Zhang B., Yu P., Chen M. (2024). Advances in research on the activity evaluation, mechanism and structure-activity relationships of natural antioxidant peptides. Antioxidants (Basel).

[150] Gao R., Yu Q., Shen Y., Chu Q., Chen G., Fen S., Yang M., Yuan L., McClements D.J., Sun Q. (2021). Production, bioactive properties, and potential applications of fish protein hydrolysates: developments and challenges. Trends Food Sci. Technol..

[151] Wang L., Li P., Zheng F., Zhu Z., Bai F., Gao R. (2024). Collagen peptides from sturgeon swim bladder prolong the lifespan and healthspan in *Caenorhabditis elegans*. J. Sci. Food Agric..

[152] Ali Z., Wang Z., Amir R.M., Younas S., Wali A., Adowa N., Ayim I. (2016). Potential uses of vinegar as a medicine and related in vivo mechanisms. Int. J. Vitam. Nutr. Res..

[153] Zhou T., Wang N., Xue Y., Ding T., Liu X., Mo X., Sun J. (2016). Electrospun tilapia collagen nanofibers accelerating wound healing via inducing keratinocytes proliferation and differentiation. Colloids Surf. B Biointerfaces.

[154] Davison-Kotler E., Marshall W.S., García-Gareta E. (2019). Sources of collagen for biomaterials in skin wound healing. Bioengineering.

[155] Huang J.-Y., Wong T.-Y., Tu T.-Y., Tang M.-J., Lin H.-H., Hsueh Y.-Y. (2024). Assessment of tilapia skin collagen for biomedical research applications in comparison with mammalian collagen. Molecules.

[156] Abdelhedi O., Nasri R., Mora L., Toldrá F., Nasri M., Jridi M. (2017). Collagenous proteins from black-barred halfbeak skin as a source of gelatin and bioactive peptides. Food Hydrocoll..

[157] Fan L., Ren Y., Emmert S., Vučković I., Stojanovic S., Najman S., Schnettler R., Barbeck M., Schenke-Layland K., Xiong X. (2023). The use of collagen-based materials in bone tissue engineering. Int. J. Mol. Sci..

[158] Geahchan S., Baharlouei P., Rahman M.A. (2022). Marine collagen: a promising biomaterial for wound healing, skin anti-aging, and bone regeneration. Mar. Drugs.

[159] Xu R., Wu J., Zheng L., Zhao M. (2023). Undenatured type II collagen and its role in improving osteoarthritis. Ageing Res. Rev..

[160] Rahman M.A. (2019). Collagen of extracellular matrix from marine invertebrates and its medical applications. Mar. Drugs.

[161] Safari E., Hassan Z.M., Moazzeni S.M. (2015). Shark cartilage 14 kDa protein as a dendritic cells activator. Immunopharmacol. Immunotoxicol..

[162] Purohit K., Reddy N., Sunna A. (2024). Exploring the potential of bioactive peptides: from natural sources to therapeutics. Int. J. Mol. Sci..

[163] Qiao Q.-Q., Luo Q.-B., Suo S.-K., Zhao Y.-Q., Chi C.-F., Wang B. (2022). Preparation, characterization, and cytoprotective effects on HUVECs of fourteen novel angiotensin-I-converting enzyme inhibitory peptides from protein hydrolysate of tuna processing by-products. Front. Nutr..

[164] Chandrasekaran P., Weiskirchen R. (2024). The role of obesity in type 2 diabetes mellitus-an overview. Int. J. Mol. Sci..

[165] Bougatef H., Sila A., Bougatef A., Martínez-Alvarez O. (2024). Protein hydrolysis as a way to valorise squid-processing byproducts: obtaining and identification of ACE, DPP-IV and PEP inhibitory peptides. Mar. Drugs.

